# Supplementary material for: Thrombospondin-3 augments injury-induced cardiomyopathy by intracellular integrin inhibition and sarcolemmal instability
Source: Nat Commun. 2019 Jan 8;10:76. doi: 10.1038/s41467-018-08026-8 (PMC6325143; doi:10.1038/s41467-018-08026-8)
Supplement: Supplementary file 1 — Supplementary Information [file 41467_2018_8026_MOESM1_ESM.pdf]

Supplementary Information

Thrombospondin-3 augments injury-induced cardiomyopathy by intracellular integrin inhibition and sarcolemmal instability

Tobias G. Schips,[1] Davy Vanhoutte,[1] Alexander Vo,[1] Robert N. Correll,[1] Matthew J. Brody,[1] Hadi Khalil,[1] Jason Karch,[1,2] Andoria Tjondrokoesoemo, [1] Michelle A. Sargent,[1] Marjorie Maillet,[1] Robert S Ross,[3] Jeffery D. Molkentin[1, 2,]*

[1] Department of Pediatrics, Cincinnati Children's Hospital Medical Center, Cincinnati, OH, 45229, USA.

[2] Howard Hughes Medical Institute, Cincinnati Children's Hospital Medical Center, Cincinnati, OH, 45229, USA.

[3] Division of Cardiology, Department of Medicine, University of California at San Diego School of Medicine, La Jolla, CA 92093 USA.

Supplementary Figures 1-8

Supplementary information showing uncropped western blot gel files for all figures

Supplementary Figure 1

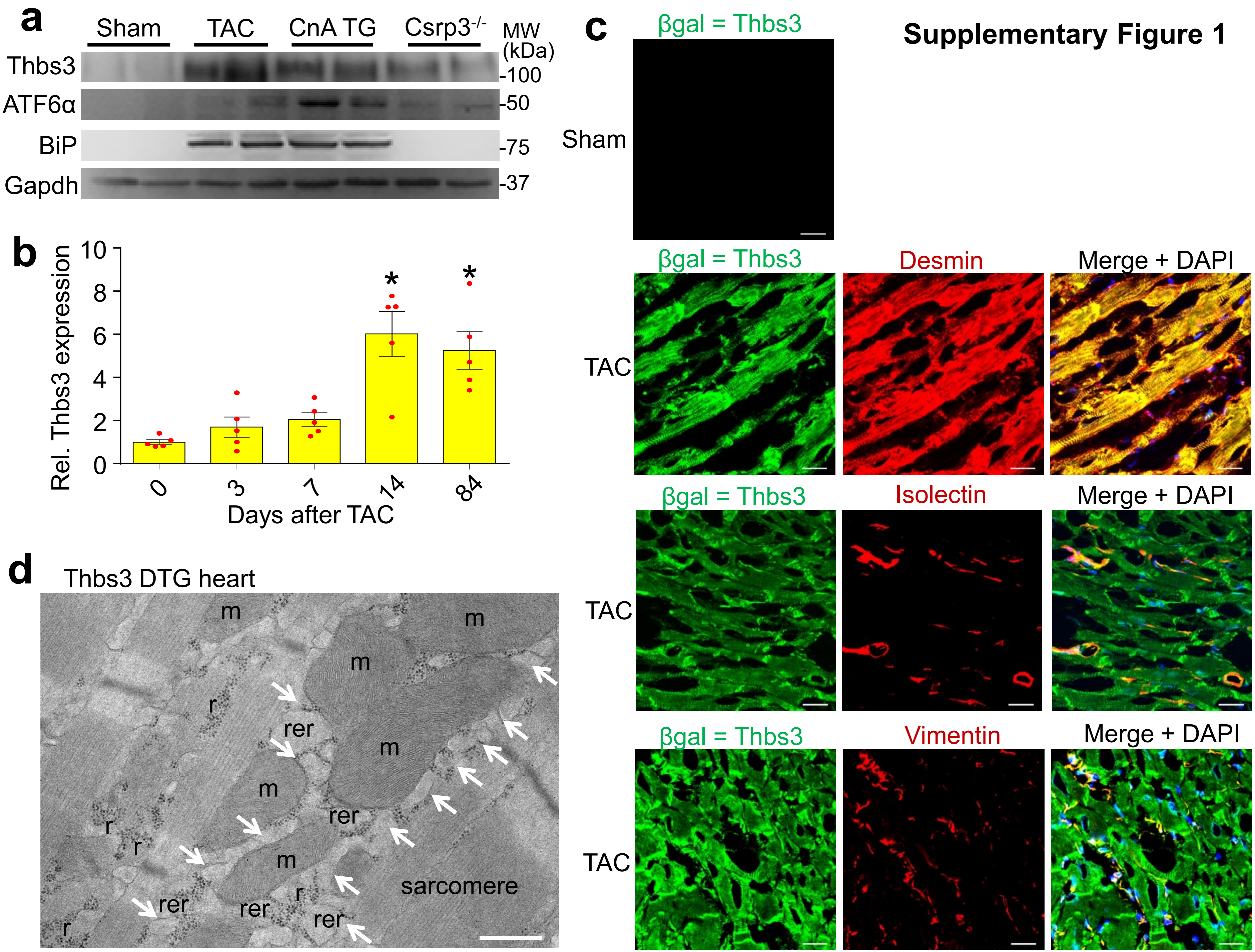

Supplementary Figure 1. *Thbs3 is expressed in the diseased heart and induces vesicular expansion of the rough ER.* **a**, Representative Western blots for Thbs3, nuclear form of ATF6α, BiP and Gapdh as loading control from heart protein extracts from mice that were sham-operated, subjected to 2 weeks of TAC, that contain the activated calcineurin A transgene (CnA) or that lack the *Csrp3* gene. **b**, Time course qRT-PCR analysis of Thbs3 mRNA expression after the indicated time point of TAC stimulation (n = 5 animals per time point) relative to Rpl13 reference gene expression. The data are represented as fold induction over sham operated controls. *P<0.05 versus sham. Statistical analysis was performed using one-way ANOVA and Turkey multiple comparisons test. Results were summed from 3 independent experiments and error bars are +/- standard error of the mean. **c**, Immunohistochemistry for β-galactosidase (β-gal = Thbs3, green), desmin (red), isolectin (red), vimentin (red) and DAPI (blue) from hearts of mice that were sham-operated or subjected to 2 weeks of TAC. Scale bars = 20 μm. **d**, Transmission electron microscopy (EM) of a heart section from a Thbs3 DTG mouse. Scale bar is 600 nm. The white arrows show expanded vesicular content in these Thbs3 DTG hearts (m = mitochondria, r = ribosome, rer = rough ER).

Supplementary Figure 2

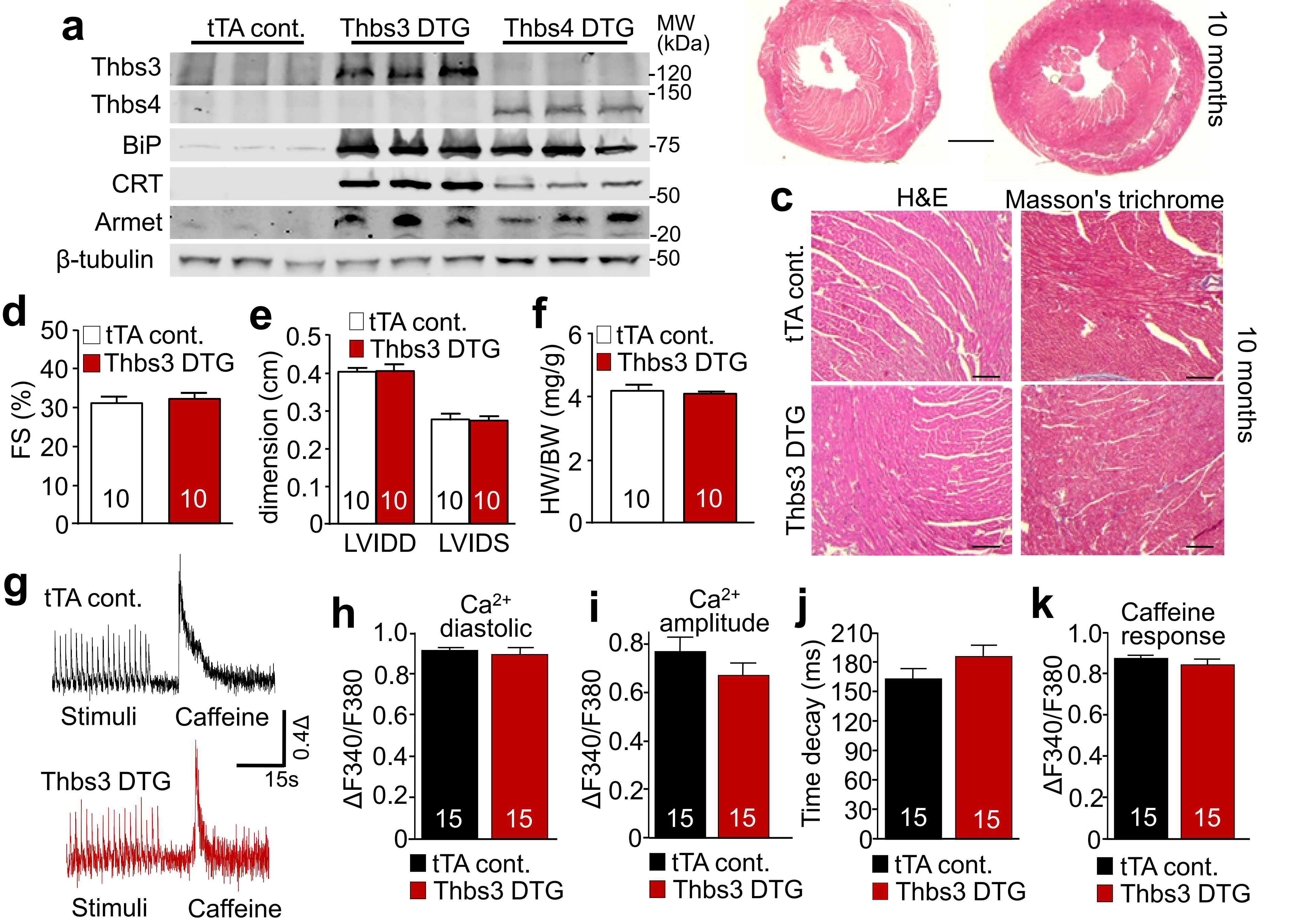

Supplementary Figure 2. *Thbs3 DTG mice show normal cardiac physiology at baseline.*
a, Western blots of heart protein extracts from tTA, Thbs3 DTG and Thbs4 DTG mice for the indicated proteins. β-tubulin serves as processing and loading control. Abbreviations: CNX, calnexin; CRT, calreticulin. **b**, Low magnification images of tTA control and Thbs3 DTG cardiac sections stained with Masson's trichrome at 10 months of age. Scale bar = 1 mm **c**, High magnification images of hematoxylin and eosin (H&E) and Masson's trichrome-stained heart sections showing no overt pathology at 10 months of age. Scale bars = 100 μm **d**, Fractional shortening (FS) percentage and (**e**) left ventricular dimensions in diastole (LVIDD) and systole (LVIDS) as determined by echocardiography in the indicated groups of mice at 10 months of age. **f**, Heart weight-to-body weight (HW/BW) ratios at 10 months of age. The number of mice analyzed is shown in the graphs. No statistical differences were determined between the groups. **g**, Representative Ca^{2+} traces, as well as (**h**) diastolic Ca^{2+}, (**i**) mean maximal amplitude of electrically-evoked Ca^{2+}transients and (**j**) Ca^{2+} decay time measured in isolated adult cardiomyocytes in 2 mM Ca^{2+} isolated from tTA control and Thbs3 DTG hearts at 11 weeks of age. **k**, Sarcoplasmic reticulum Ca^{2+} load measured at 0.5 mM Ca^{2+} via caffeine-induced Ca^{2+} release. Number of myocytes analyzed is shown in the histograms in panels **g-k**, which were isolated from 3 animals for each condition. No statistical differences were determined between the groups using two-tailed students T-test. Number of independent experiments are shown in each of the graphs and error bars are +/- standard error of the mean.

Supplementary Figure 3

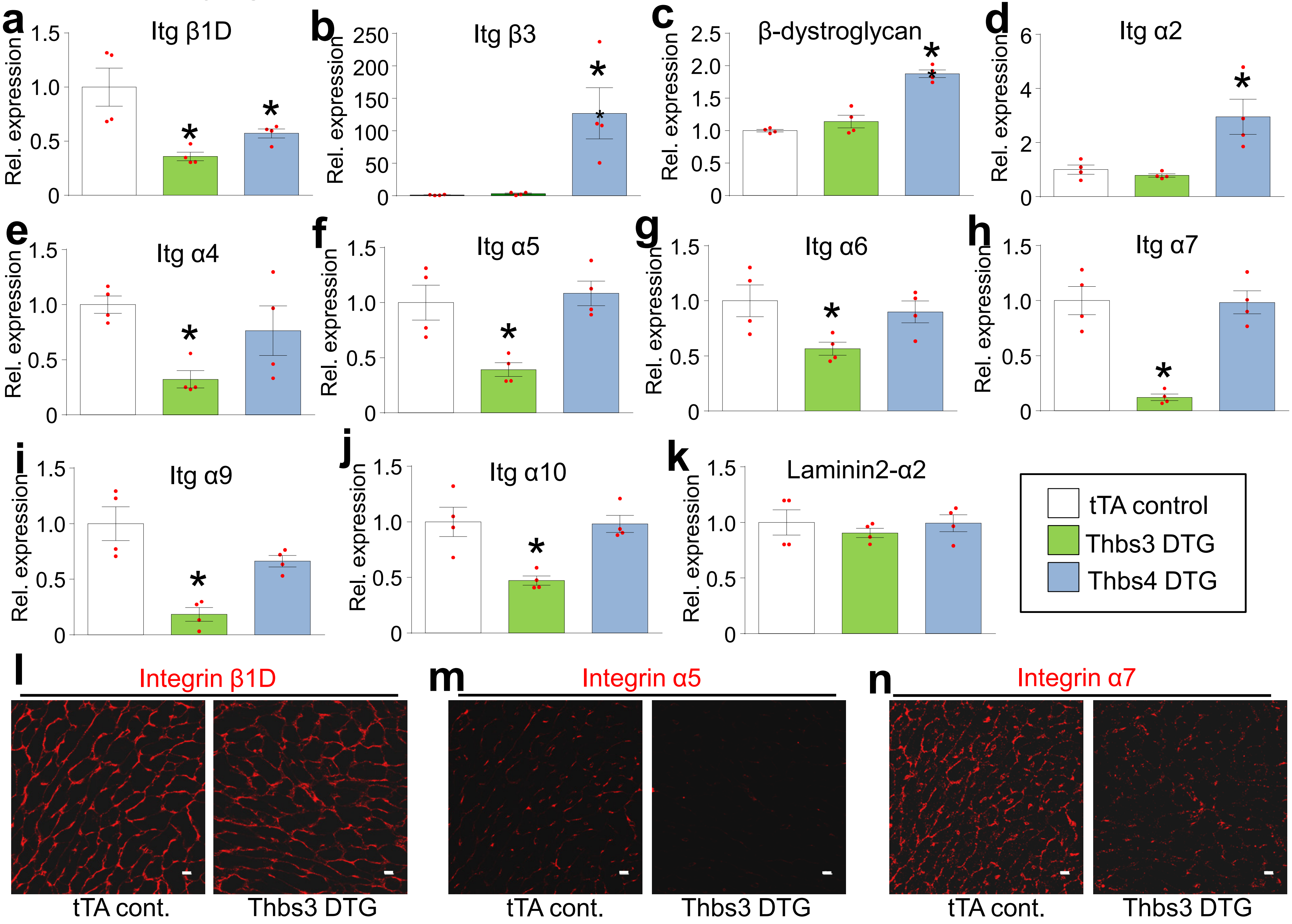

Supplementary Figure 3. *Thbs3 reduces cell surface integrin levels.* **a-k**, Quantitative analysis of protein band intensities (representative images presented in Figure 3a) from heart sarcolemma protein extracts from tTA, Thbs3 DTG and Thbs4 DTG mice for the indicated proteins (n = 4 animals per genotype). The data are represented as fold expression over tTA controls relative to Cacna1c expression. *$P<0.05$ versus tTA control. Statistical analysis was performed using one-way ANOVA and Turkey multiple comparisons test. **l-n** Immunohistochemistry for β1D integrin (**l**), α5 integrin (**m**) and α5 integrin (**n**) from hearts of tTA control and Thbs3 DTG mice. Scale bars = 10 μm. Results were summed from 4 independent experiments and error bars are +/- standard error of the mean.

Supplementary Figure 4

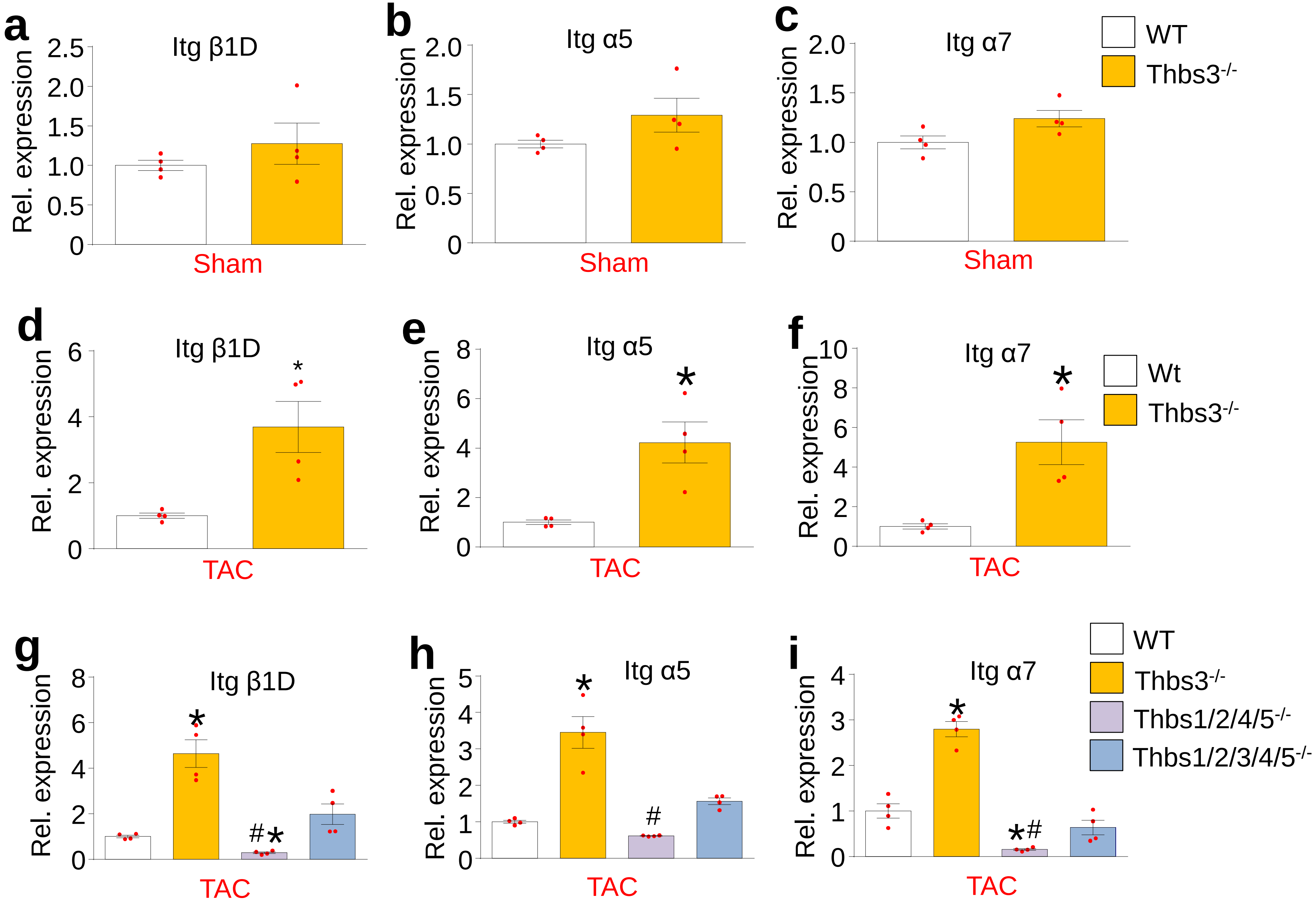

Supplementary Figure 4. *Loss of Thbs3 increases cell surface integrin levels after injury.*
a-f, Quantitative analysis of protein band intensities (representative images presented in Figure 4g) of indicated integrin proteins from heart sarcolemma protein extracts from WT and *Thbs3*$^{-/-}$ mice 12 weeks after sham (**a-c**) or TAC (**d-f**) surgery (n = 4 animals per genotype). The data are represented as fold expression over WT controls relative to Cacna1c expression. *P<0.05 versus WT control. Statistical analysis was performed using two-tailed students T-test. **g-i**, Quantitative analysis of protein band intensities (representative images presented in Figure 5g) of the indicated integrin proteins from heart sarcolemma protein extracts from WT, *Thbs3*$^{-/-}$, *Thbs1/2/4/5*$^{-/-}$, and *Thbs1/2/3/4/5*$^{-/-}$ mice 1 week after TAC surgery (n = 4 animals per genotype). The data are represented as fold expression over WT controls relative to Cacna1c expression. *P<0.05 versus WT control, $^{\#}$P<0.05 versus *Thbs3*$^{-/-}$. Statistical analysis was performed using one-way ANOVA and Turkey multiple comparisons test. Results were summed from 4 independent experiments and error bars are +/- standard error of the mean.

Supplementary Figure 5

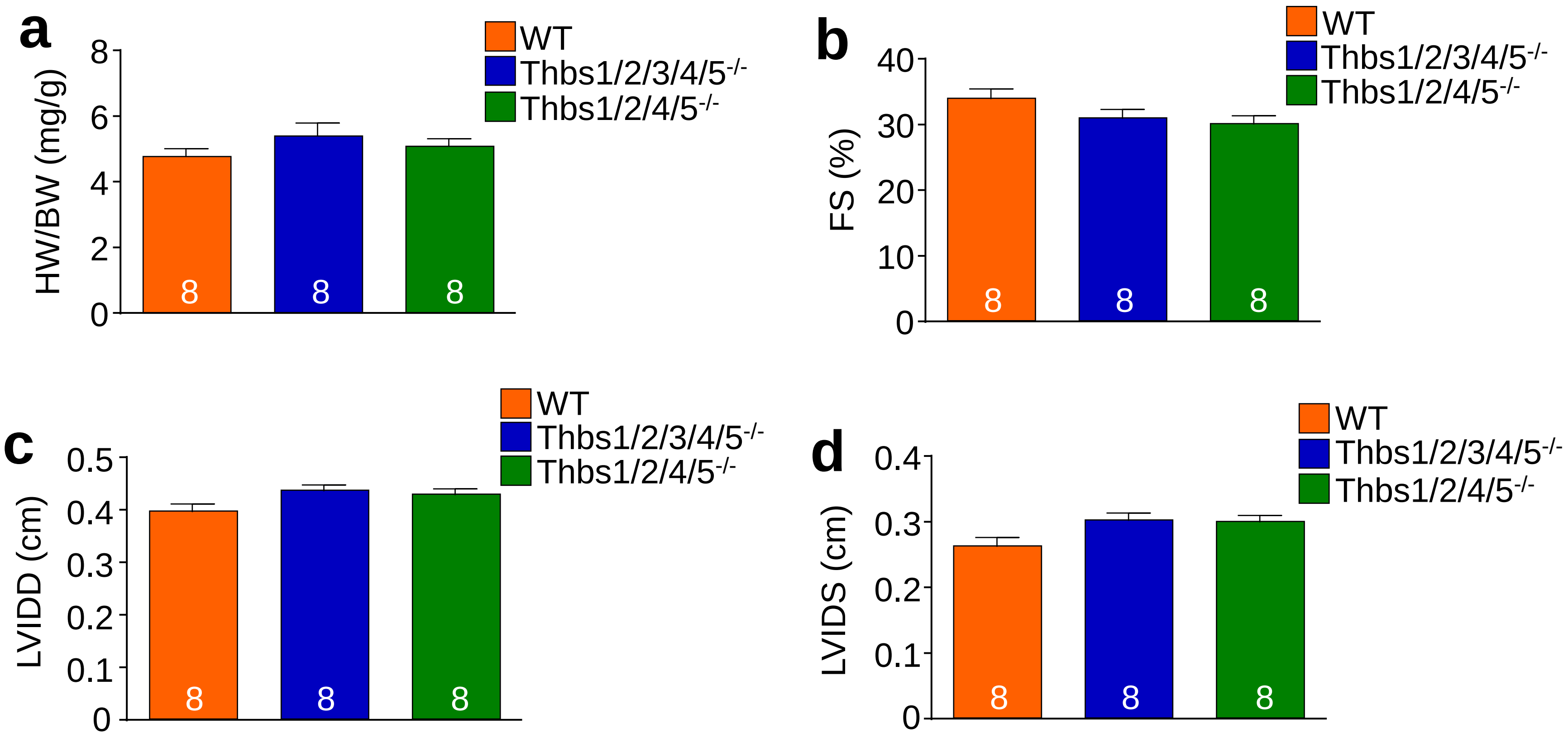

Supplementary Figure 5. *WT, Thbs1/2/4/5$^{-/-}$ and Thbs1/2/3/4/5$^{-/-}$ cardiac phenotype at 12 months of age.* **a**, Heart weight-to-body weight (HW/BW) ratios at 12 months of age. **b**, Fractional shortening (FS) percentage and (**c**) left ventricular dimensions in diastole (LVIDD) and (**d**) systole (LVIDS) as determined by echocardiography in the indicated groups of mice at 12 months of age. The number of mice analyzed is shown in the graphs. No statistical differences were determined between the groups using one-way ANOVA and Turkey multiple comparisons. Number of mice analyzed is shown in the graphs and error bars are +/- standard error of the mean.

Supplementary Figure 6

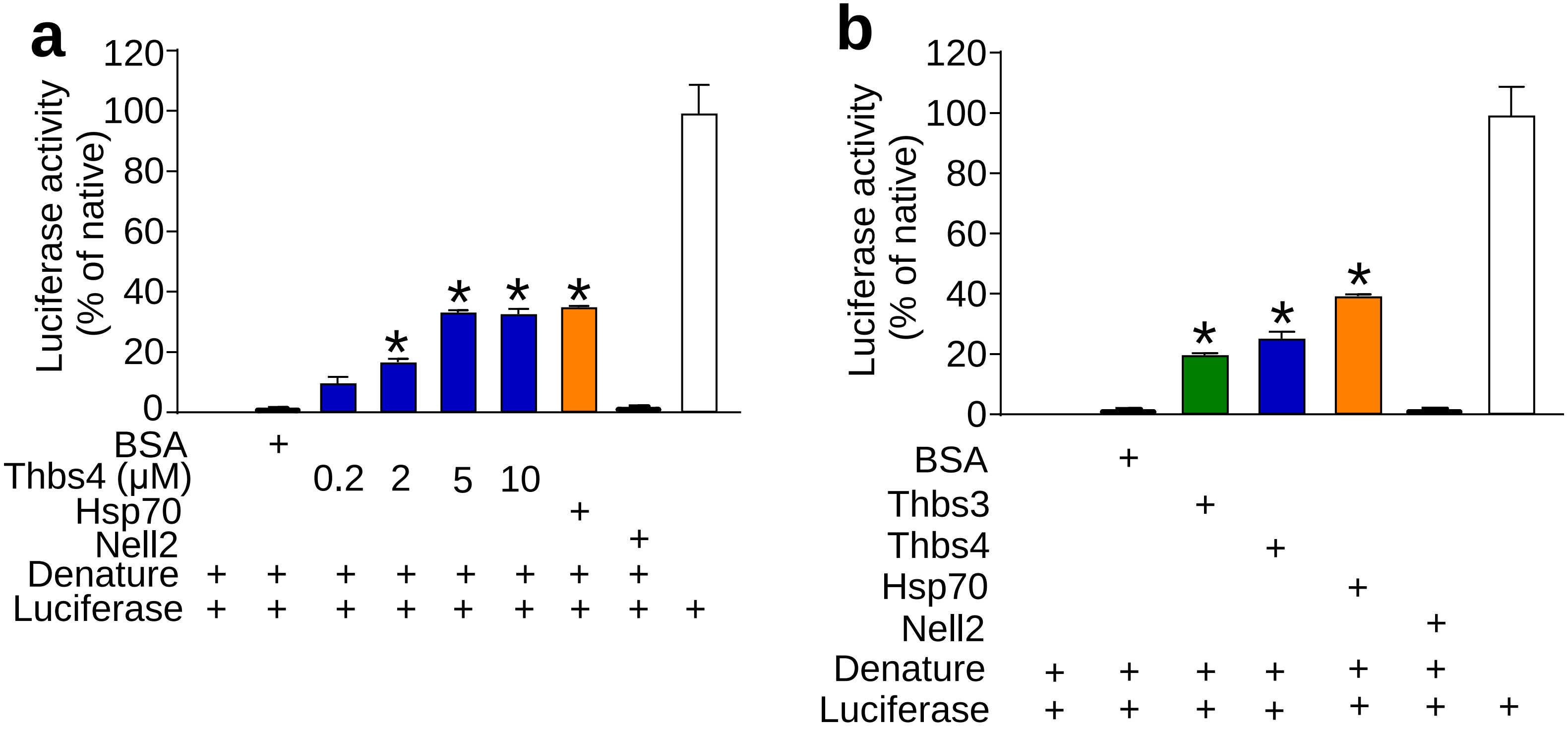

Supplementary Figure 6. *Thbs proteins have direct chaperone activity in vitro.* **a**, Denatured luciferase refolding assay using bovine serum albumin (BSA, 2 μM) as well as recombinant heat shock protein 70 (Hsp70, 2 μM), recombinant Nell2 (2 μM) and increasing recombinant Thbs4 concentrations (0.2 μM, 2 μM, 5 μM, 10 μM). Refolded luciferase activity is expressed as percentage of native, non-denatured luciferase activity (100%). Mean ± SEM of 2 independent experiments measured in triplicate are presented; * p≤ 0.05 versus denatured luciferase. Statistical analysis was performed using one-way ANOVA and Turkey multiple comparisons test. **b**, Refolding luciferase activity expressed as a percentage of native luciferase activity in the presence of BSA (2 μM), recombinant Thbs4 (2 μM), Hsp70 (2 μM) or Thbs3 (2 μM) and Hsp70 (2 μM). Mean ± SEM of 2 independent experiments measured in triplicate are presented; * p≤ 0.0001 versus BSA. Statistical analysis was performed using one-way ANOVA and Turkey multiple comparisons test.

Supplementary Figure 7

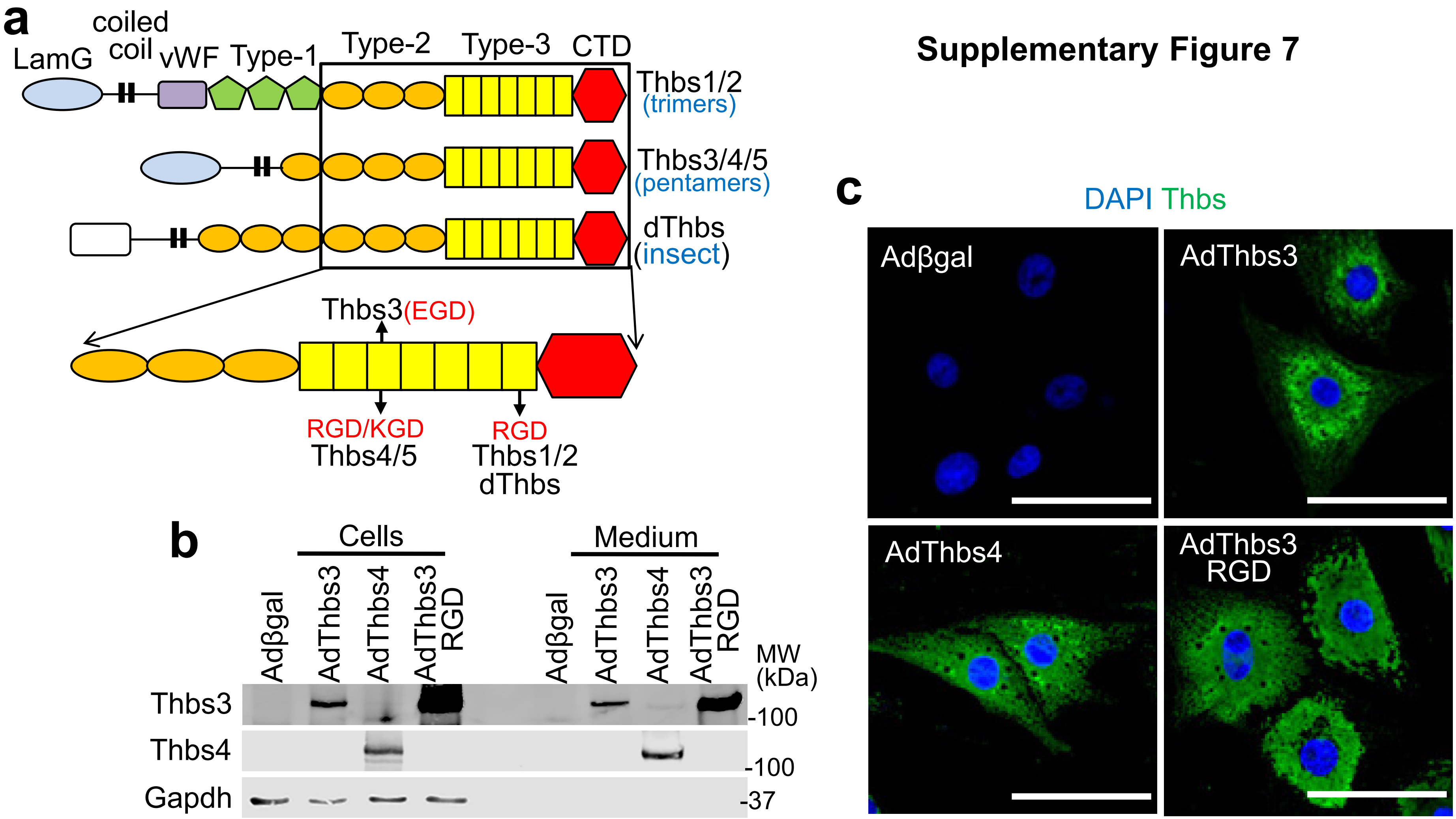

Supplementary Figure 7. *Replacing the missing integrin RGD binding domain in Thbs3.* **a**, Schematic diagram of Thbs1/2, Thbs3/4/5 and insect Thbs with their conserved domains shown. The enlarged region highlights the c-terminal integrin binding sites embedded within the Type-3 repeat domains. **b**, Representative Western blots for Thbs3 and Thbs4 from neonatal rat ventricular myocytes (NRVM) infected with Adβgal, AdThbs3, AdThbs4 and AdThbs3 RGD adenovirus. Gapdh serves as loading controls. Whole cell lysates and concentrated protein from the media are shown. The data show that Thbs3 RGD is secreted as efficiently as Thbs3 or Thbs4. **c**, Immunocytochemistry for Thbs3, Thbs4 (green) and DAPI (blue) from NRVMs infected with Adβgal, AdThbs3, AdThbs4 and AdThbs3 RGD adenovirus. Scale bars = 50 μm. The data show that the Thbs3 RGD mutant localizes intracellularly similar to Thbs3 and Thbs4.

Supplementary Figure 8

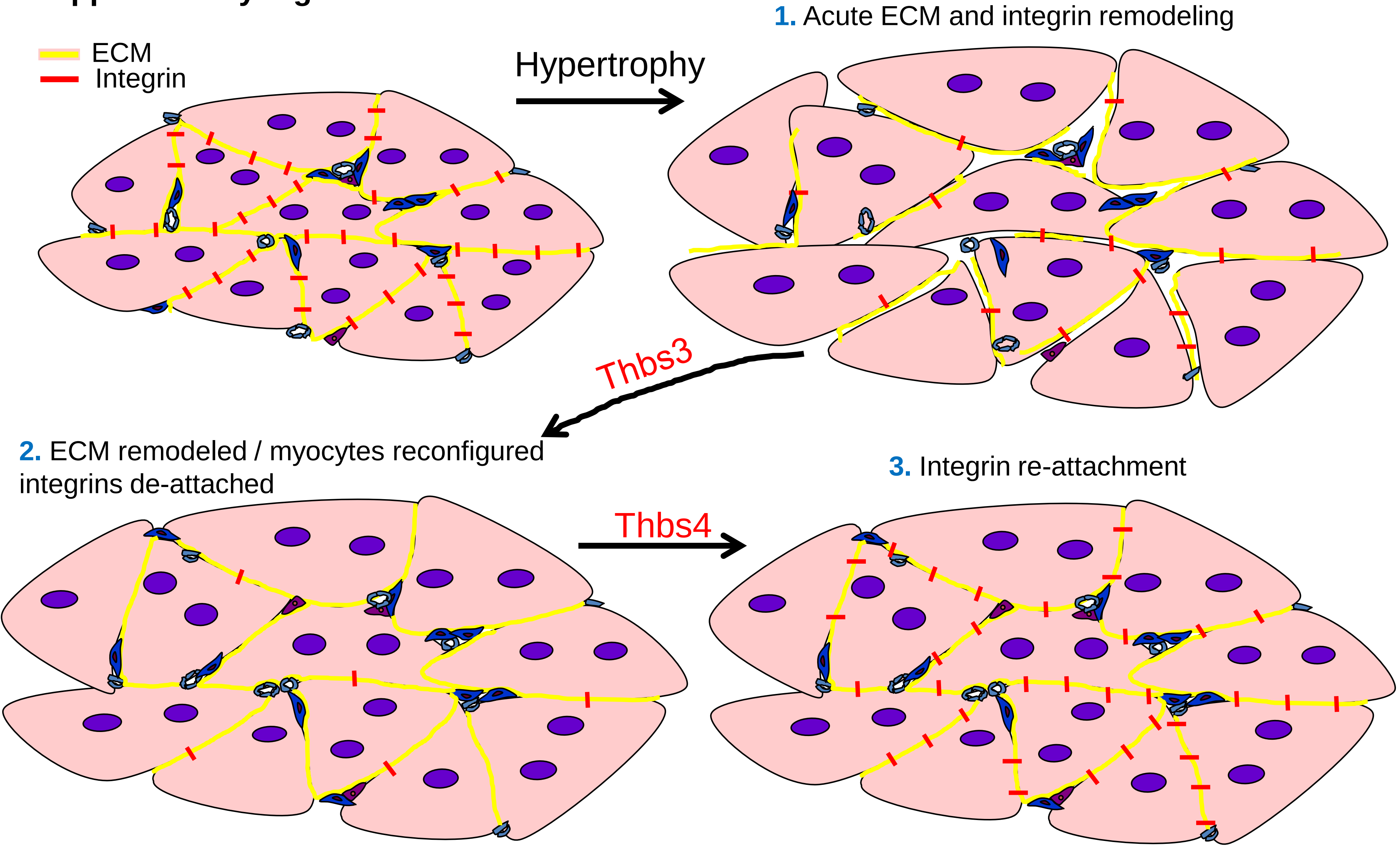

Supplementary Figure 8. *Physiologic model of Thbs3 and Thbs4 intracellular regulation of cardiomyocyte remodeling versus the ECM and integrins in hypertrophic heart tissue*. The model depicts stages where at first cardiomyocytes in the injured or hypertrophic heart remodel and lose integrin-ECM attachments through Thbs3 so the enlarged cells can be repositioned versus the ECM, yet the sarcolemma is weakened during this readjustment phase. The integrin network then re-attaches enlarged cardiomyocytes to the ECM through Thbs4 to mediate a new tissue homeostasis set point with increased membrane stability.

Supplementary Figure 9. See final page for figure legend of all uncropped blot s

Uncropped blots Supplementary Fig.1a

Thbs3

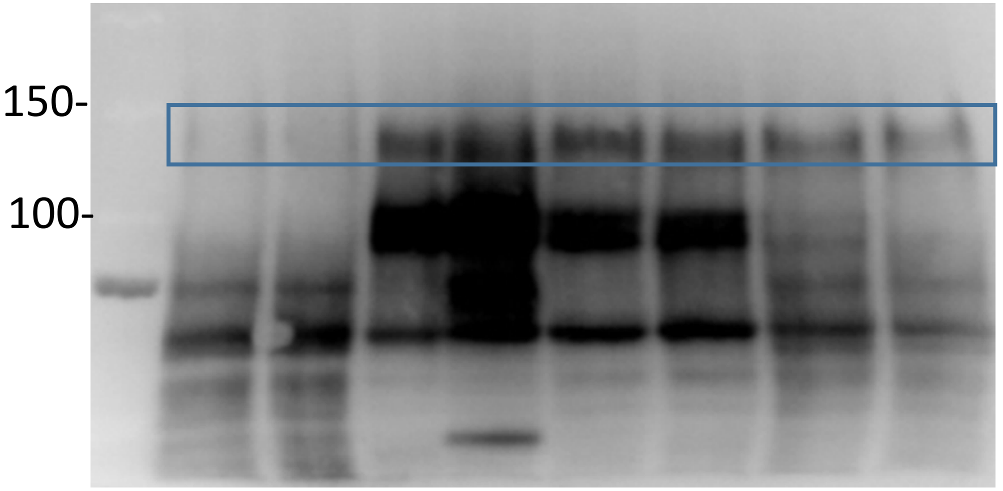

ATF6

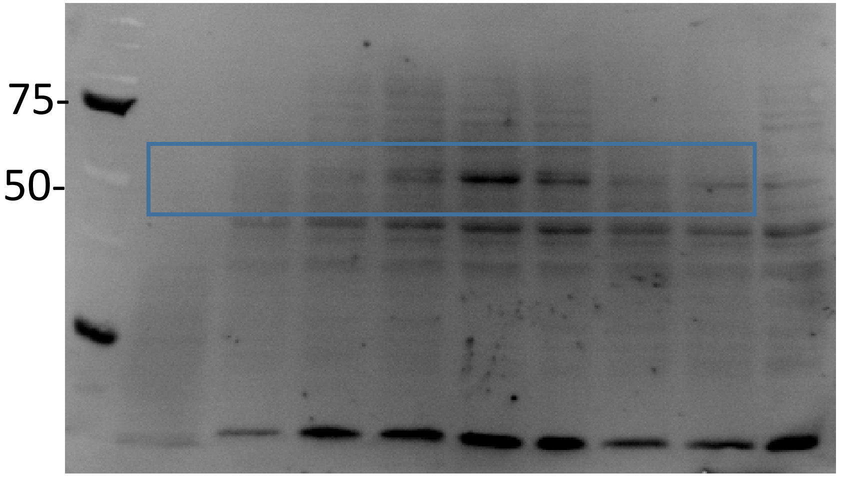

BiP

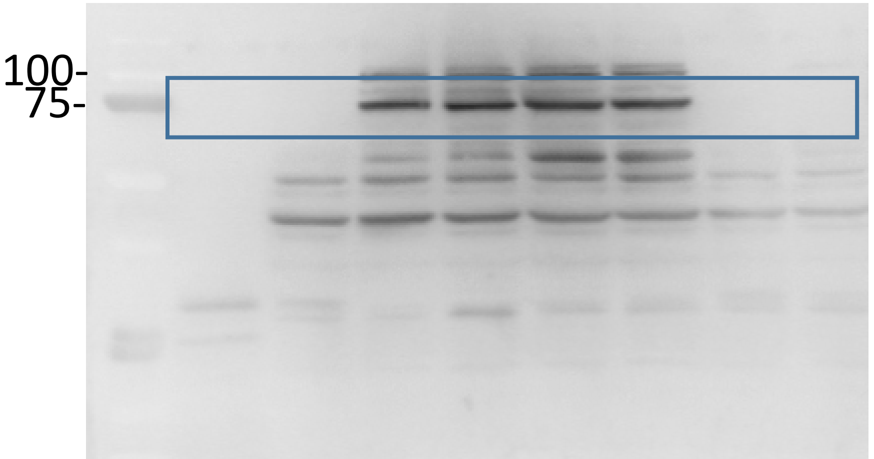

GAPDH

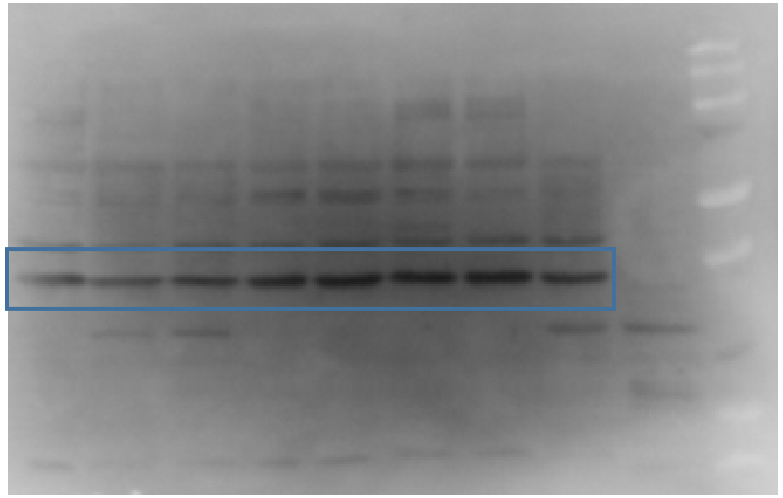

Supplementary Figure 9.

Uncropped blots Fig.1e

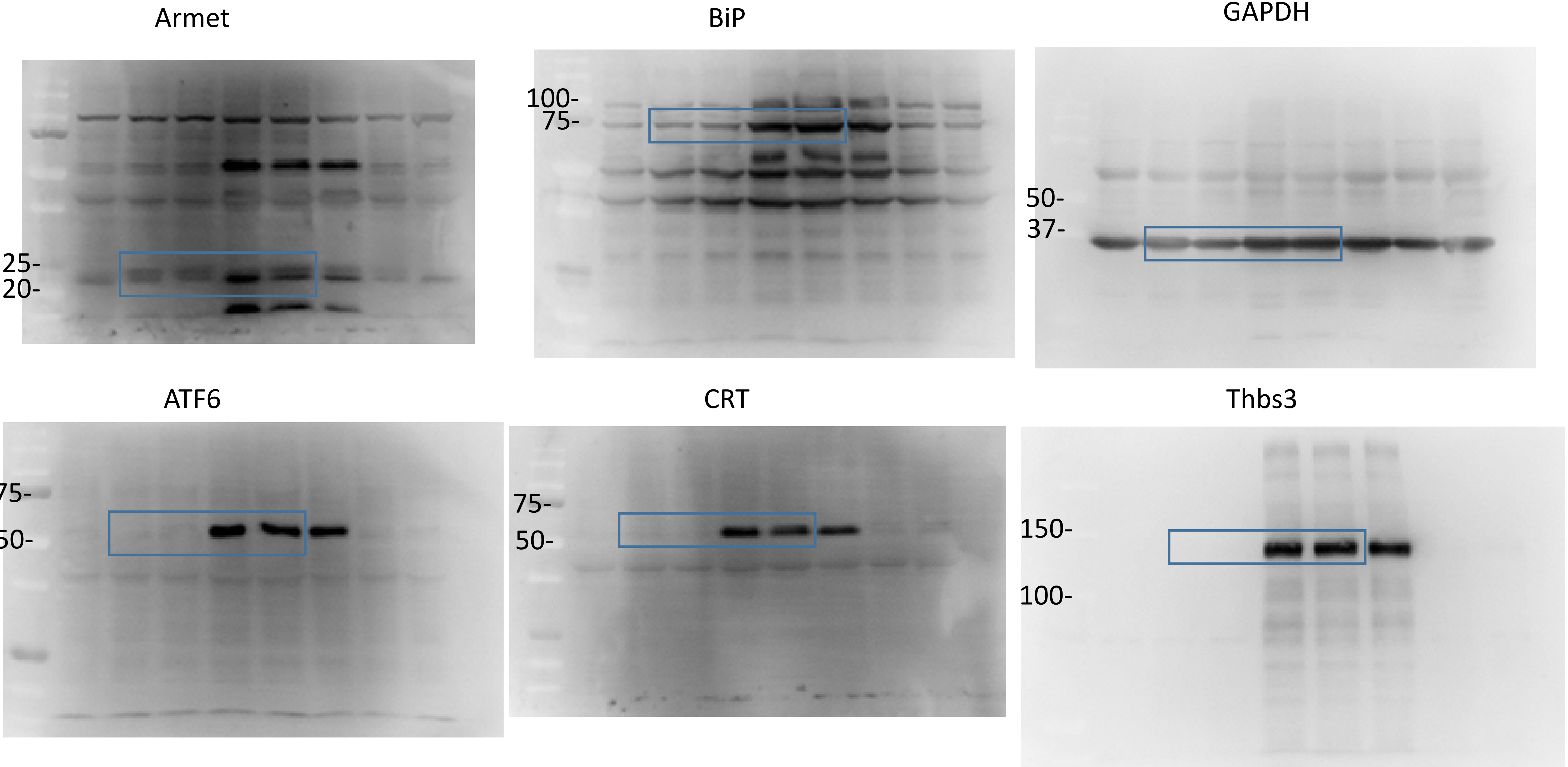

Supplementary Figure 9.

Uncropped blots Fig.1f

Input Thbs3

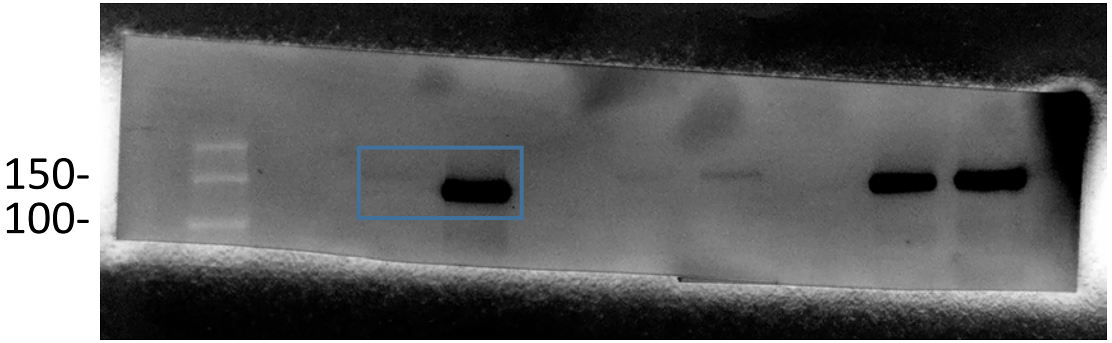

IP Myc – Blot Myc ATF6

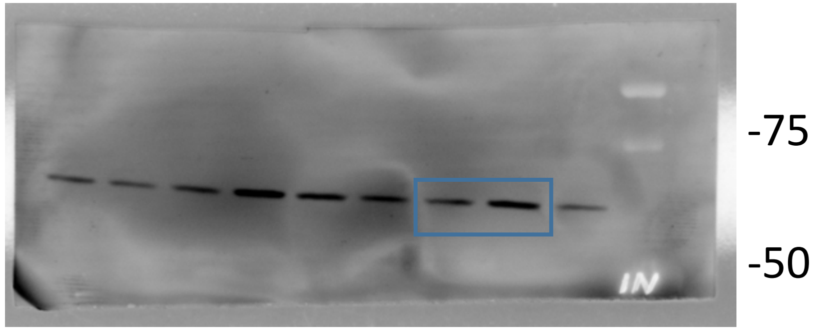

Input Myc ATF6

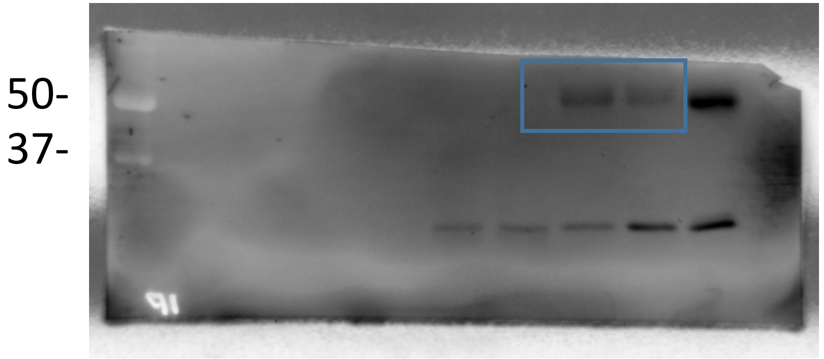

IP Myc – Blot Thbs3

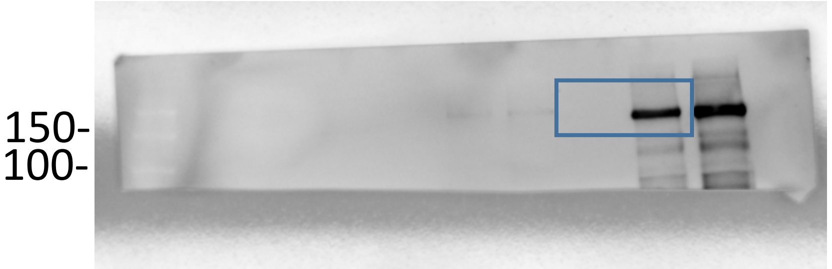

Supplementary Figure 9.

Uncropped blots Supplementary Fig.2a

Armet

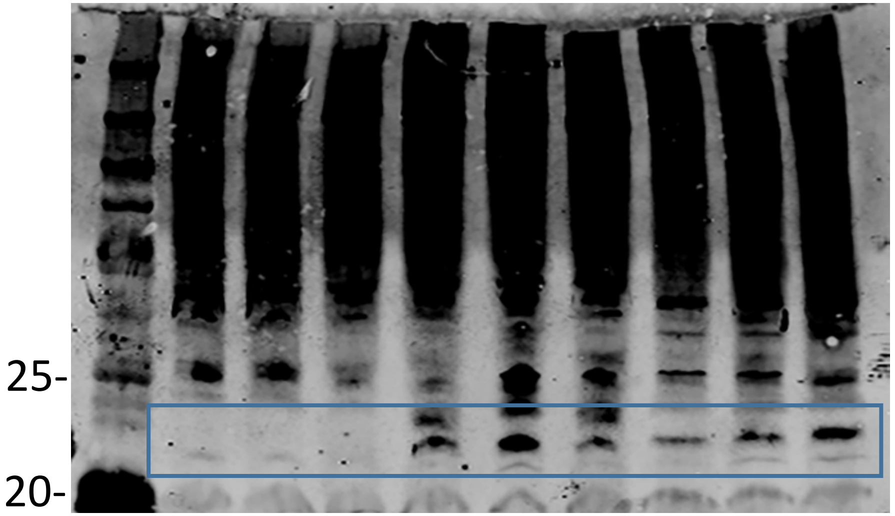

CRT

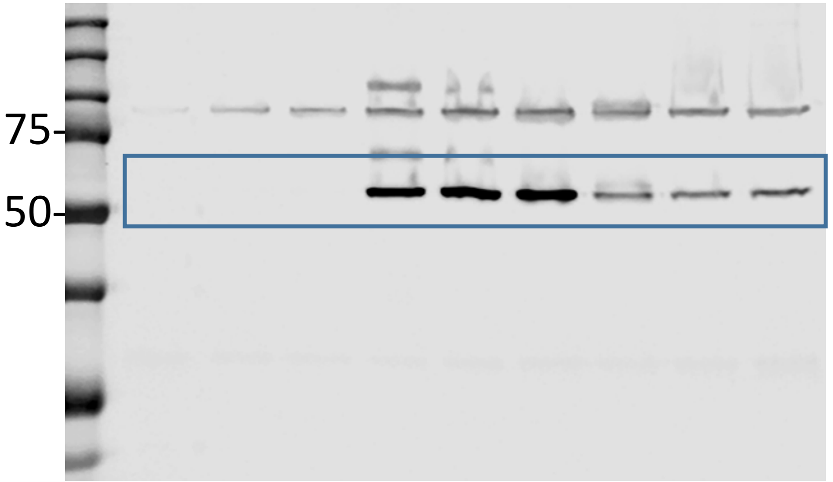

Thbs3

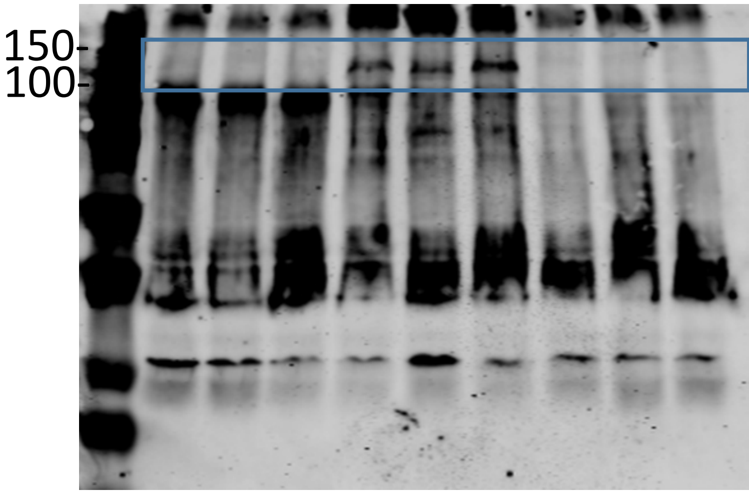

BiP

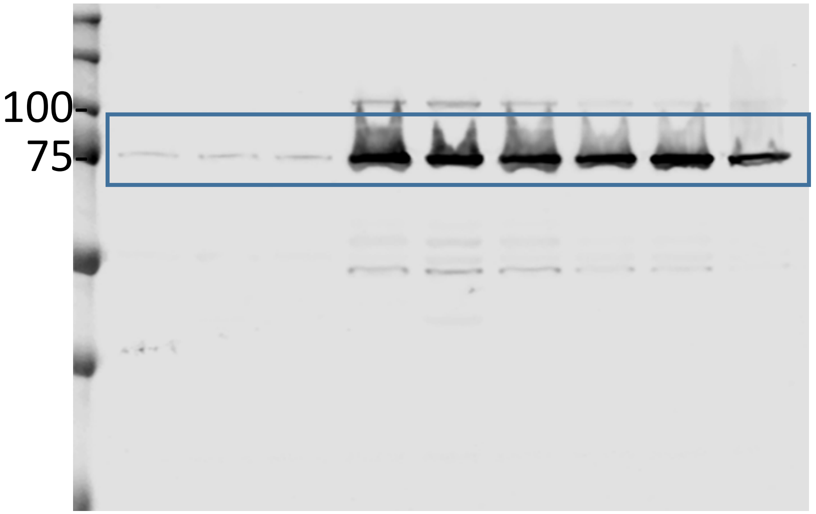

Tubulin

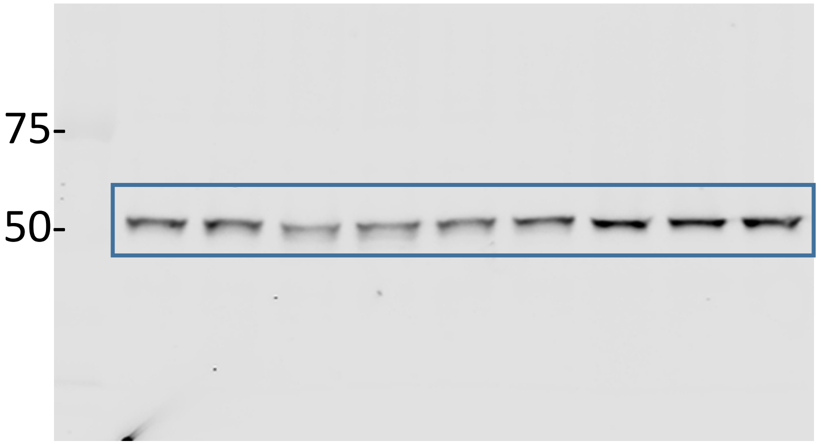

Thbs4

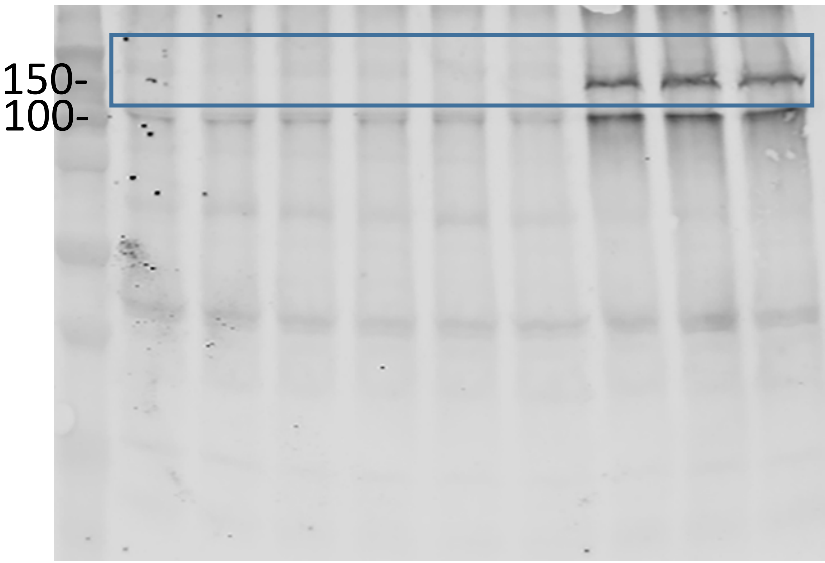

Supplementary Figure 9.

Uncropped blots Fig.3a

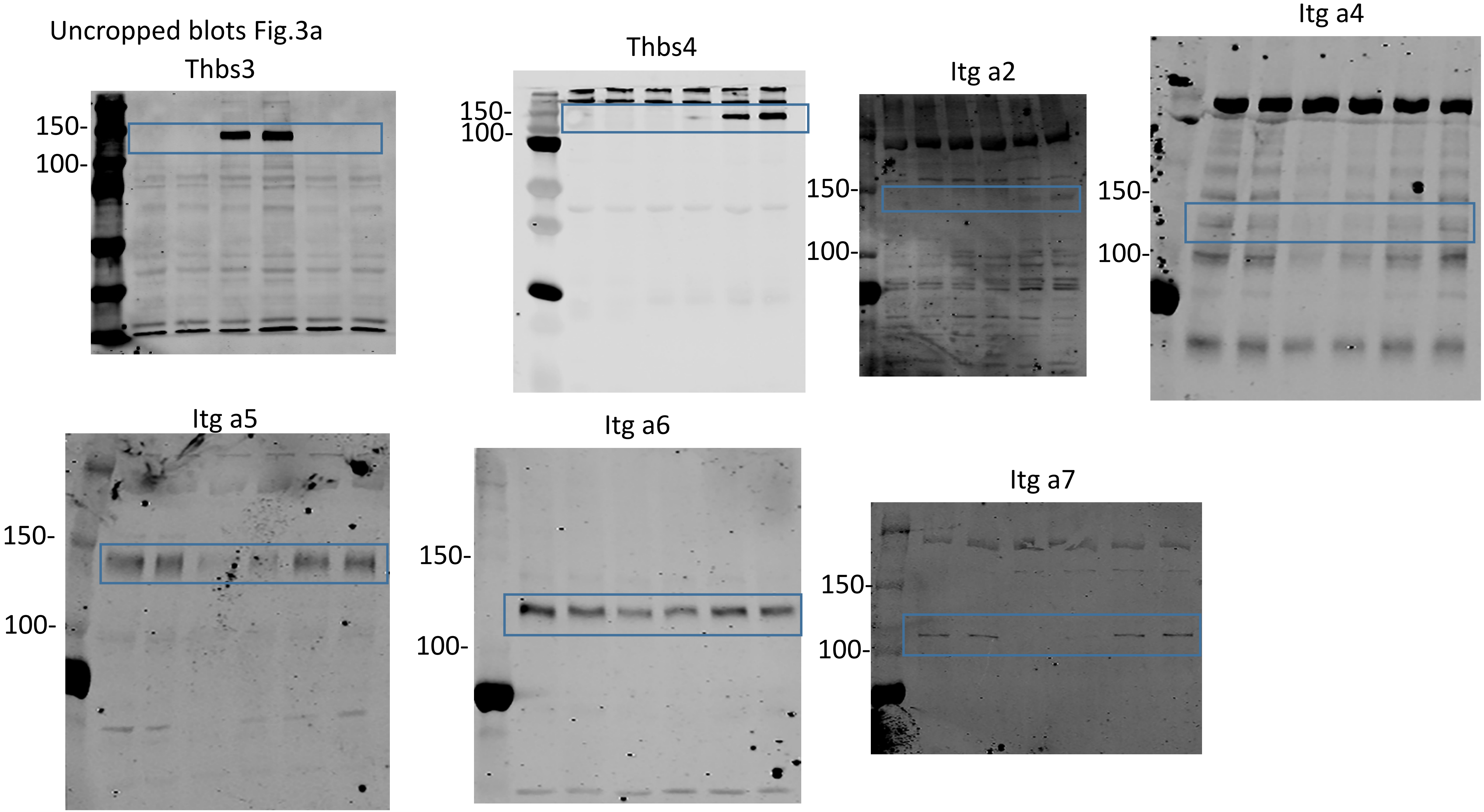

Supplementary Figure 9.

Uncropped blots Fig.3a cont.

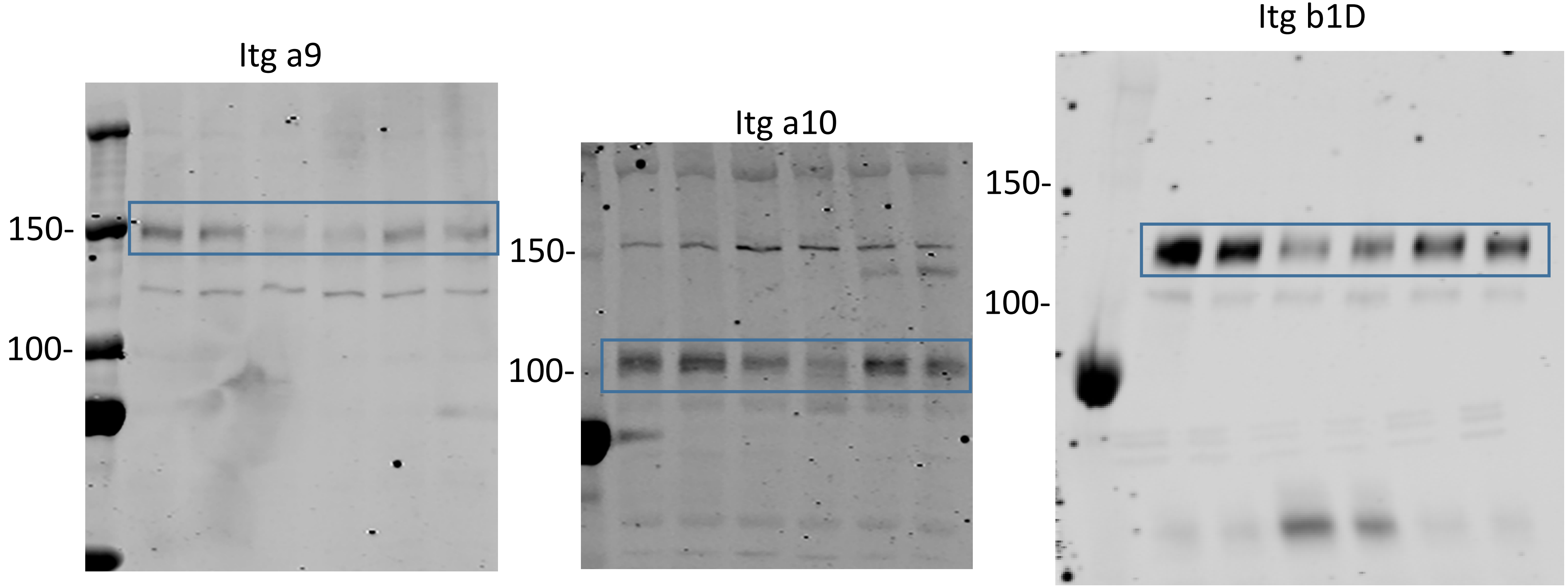

Supplementary Figure 9.

Uncropped blots Fig.3a cont.

Laminin 2

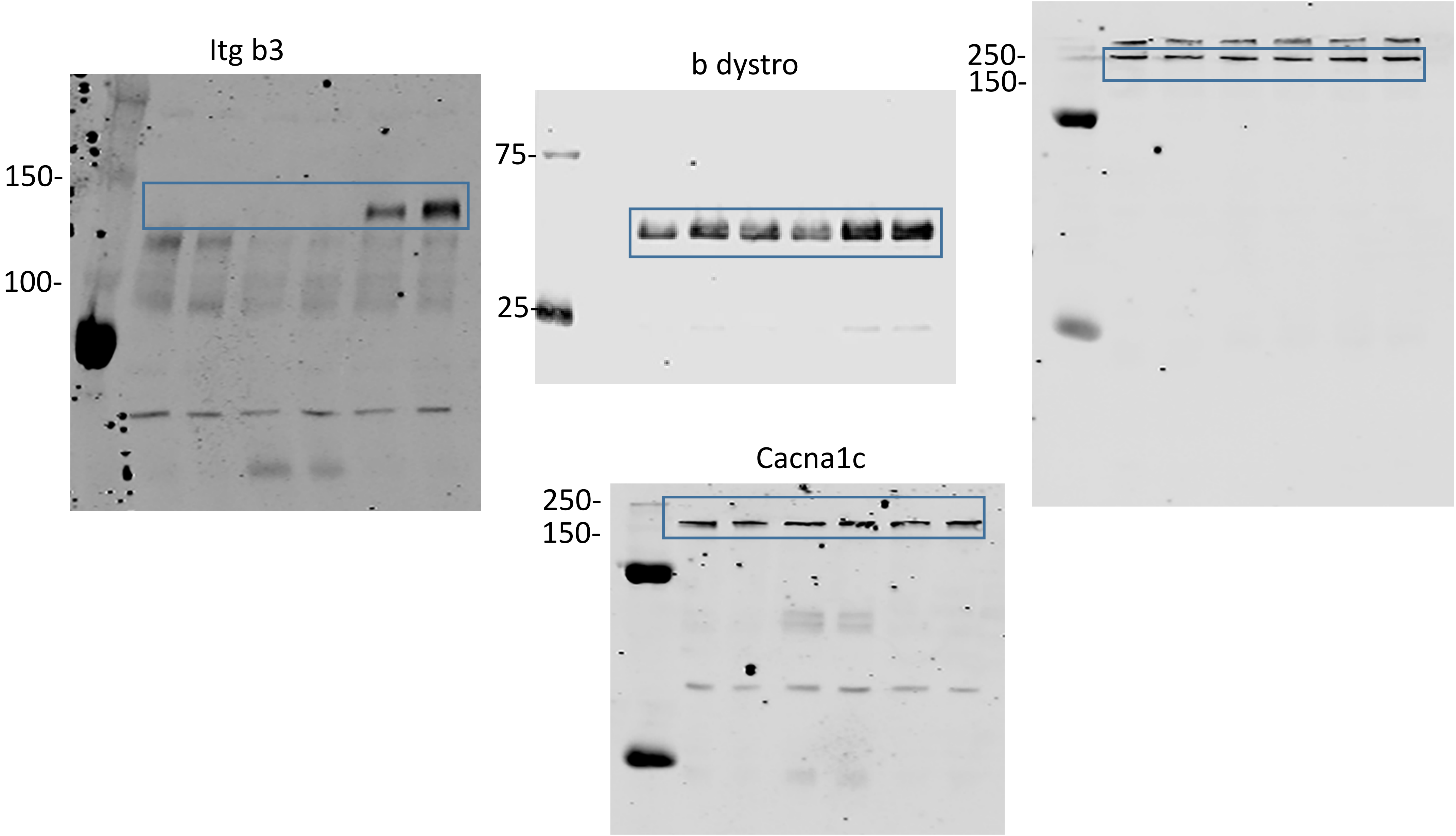

Supplementary Figure 9.

Uncropped blots Fig.3b

Itg b1D

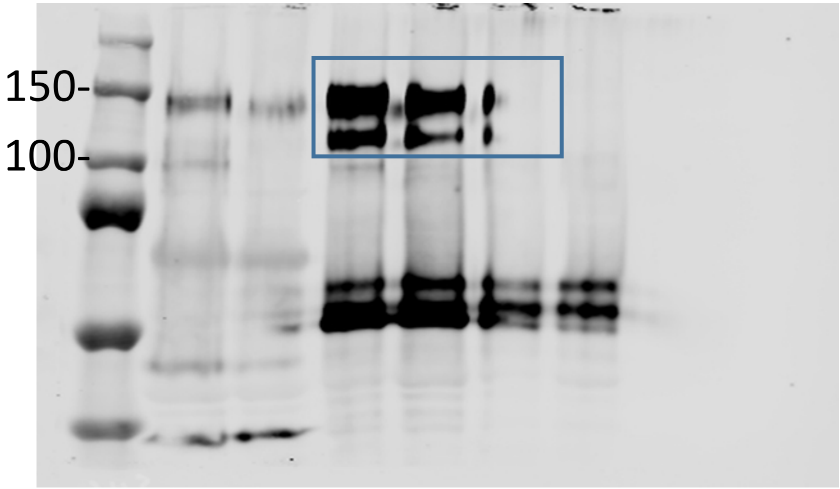

Thbs3

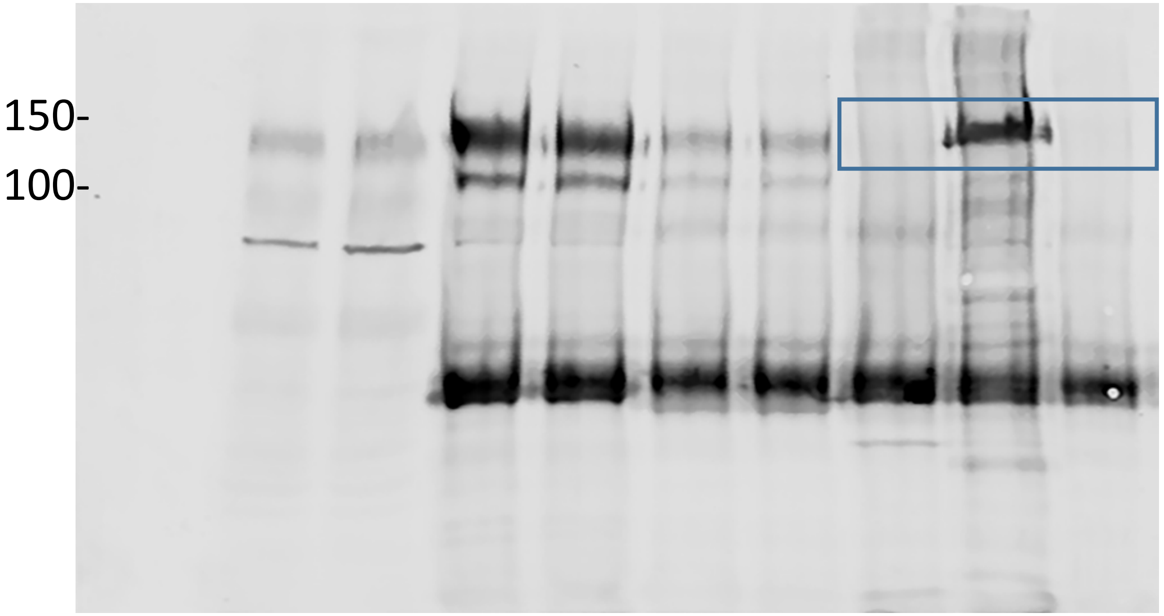

Supplementary Figure 9.

Uncropped blots Fig.3b

Input

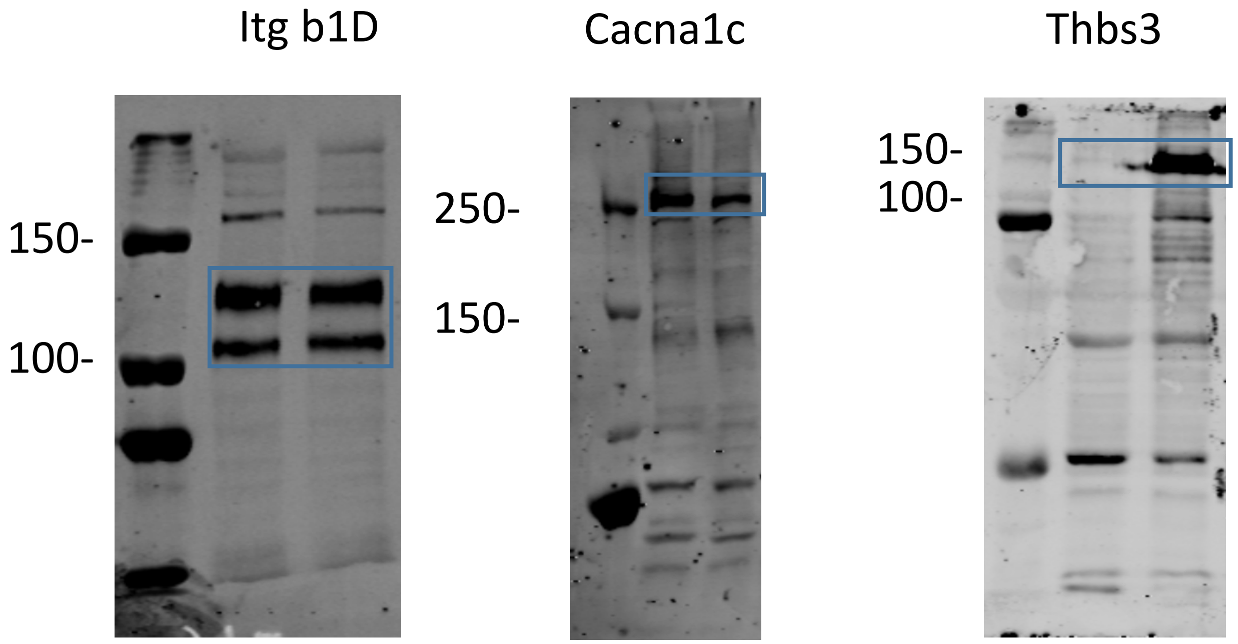

Supplementary Figure 9.

Uncropped blots Fig.4g

Sham Itg b1D

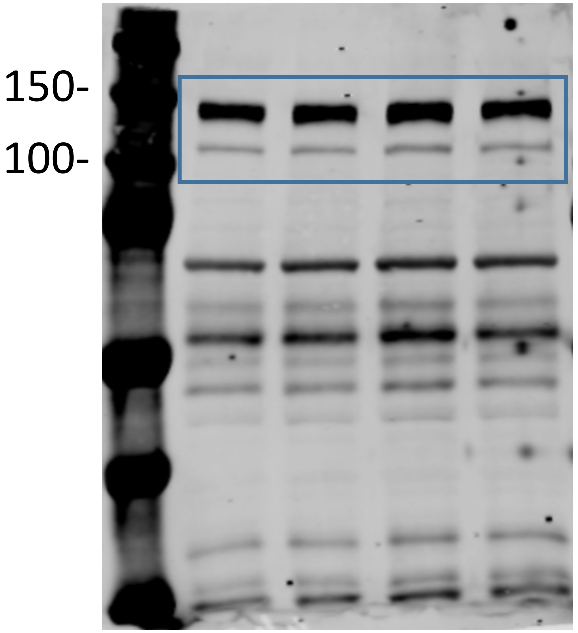

Sham Itg a5

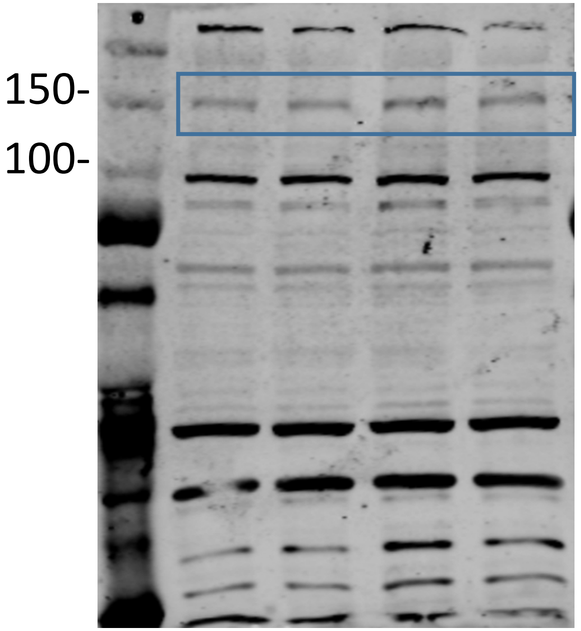

Sham Itg a7

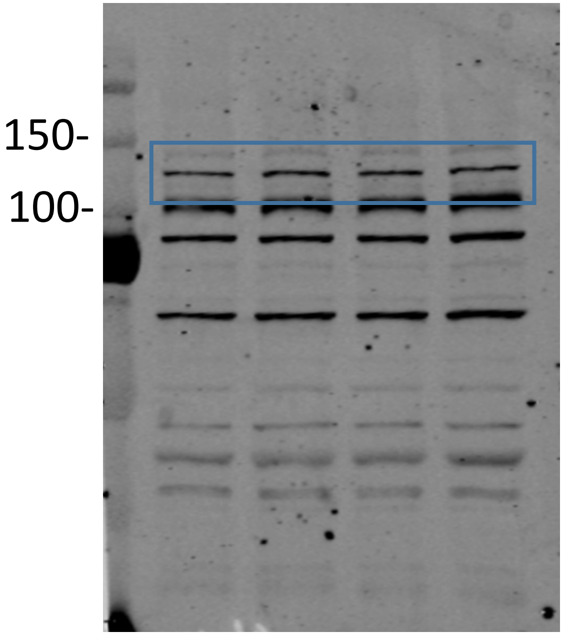

Sham Cacna1c

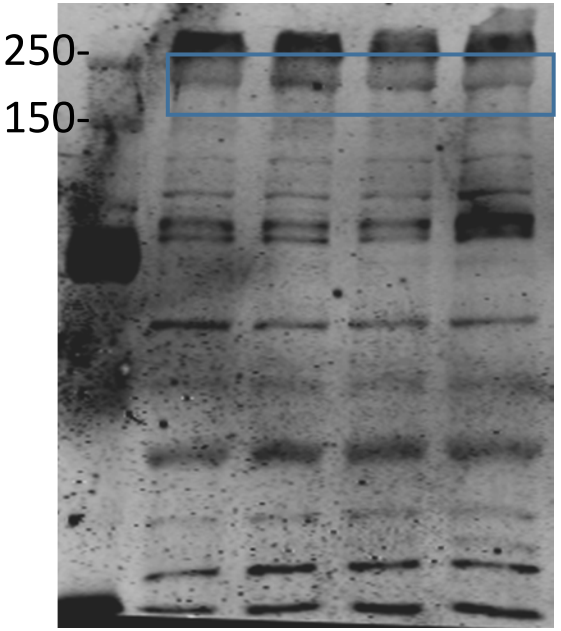

Supplementary Figure 9.

Uncropped blots Fig.4g

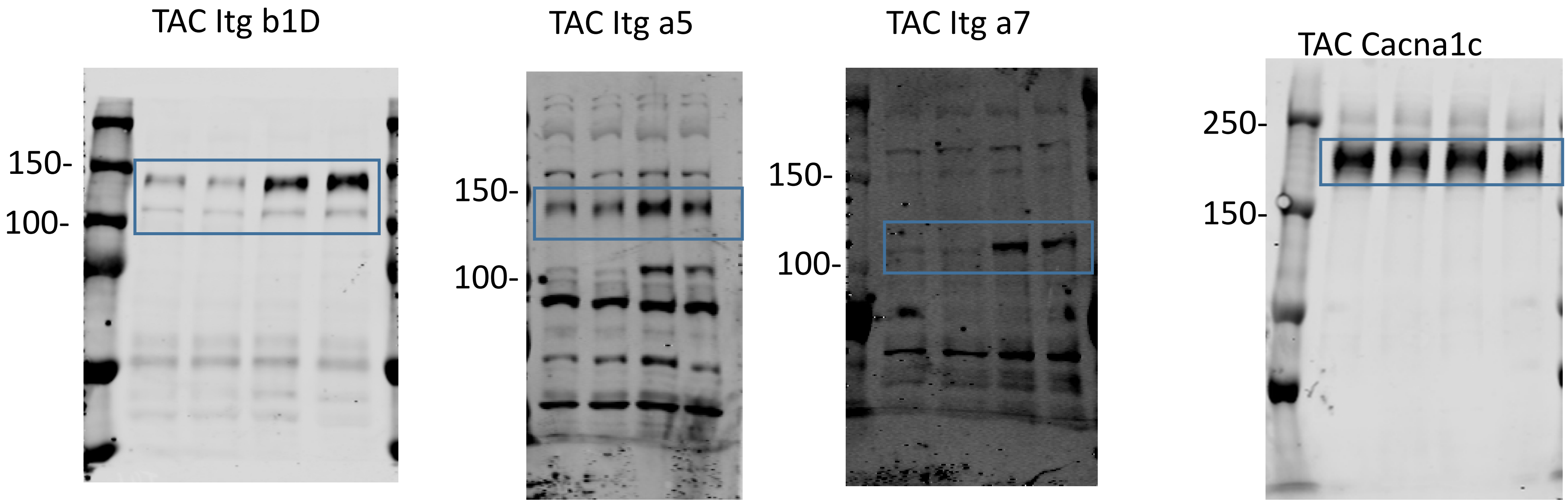

Supplementary Figure 9.

Uncropped blots Fig.5b

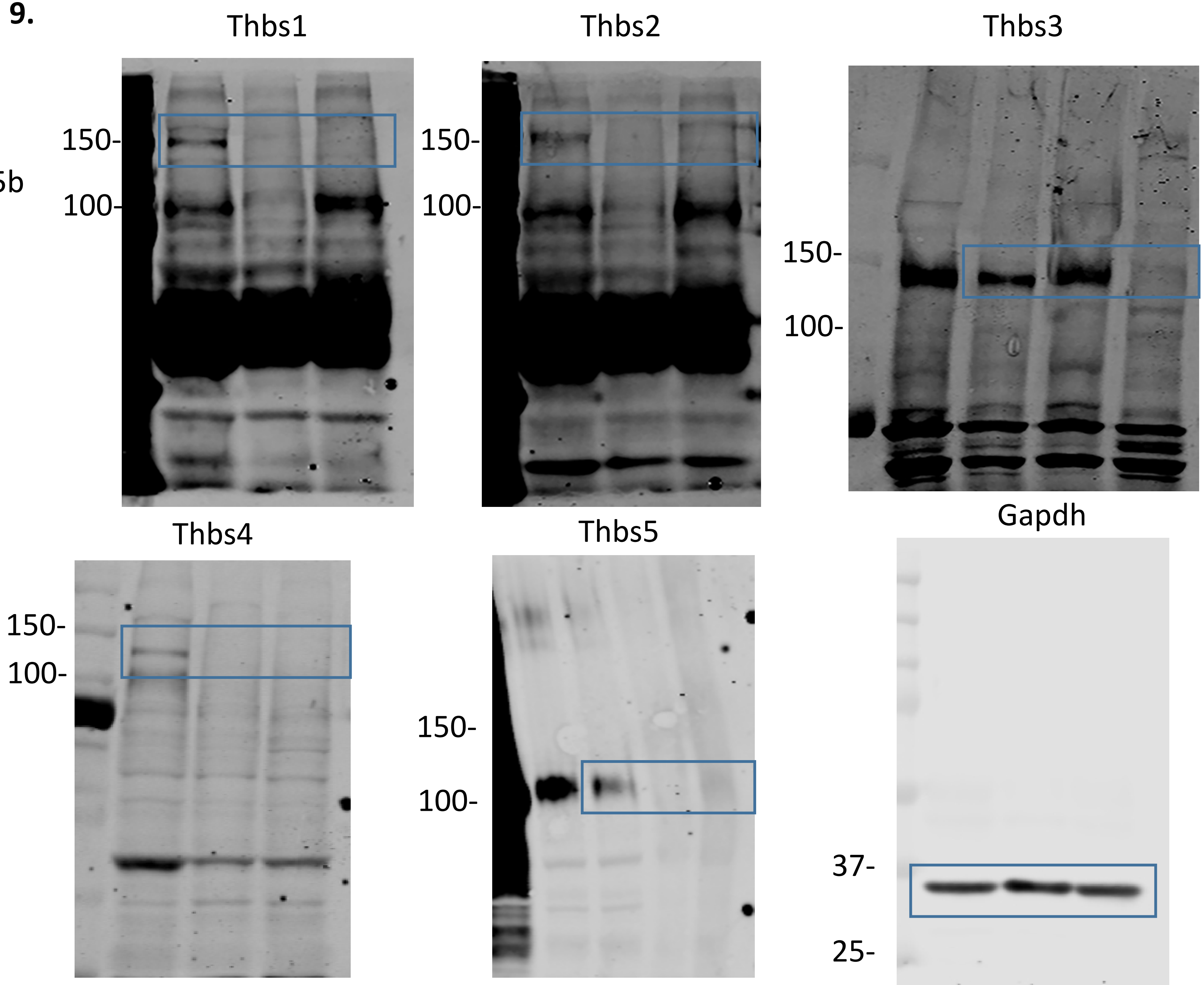

Supplementary Figure 9.

Uncropped blots Fig.5g

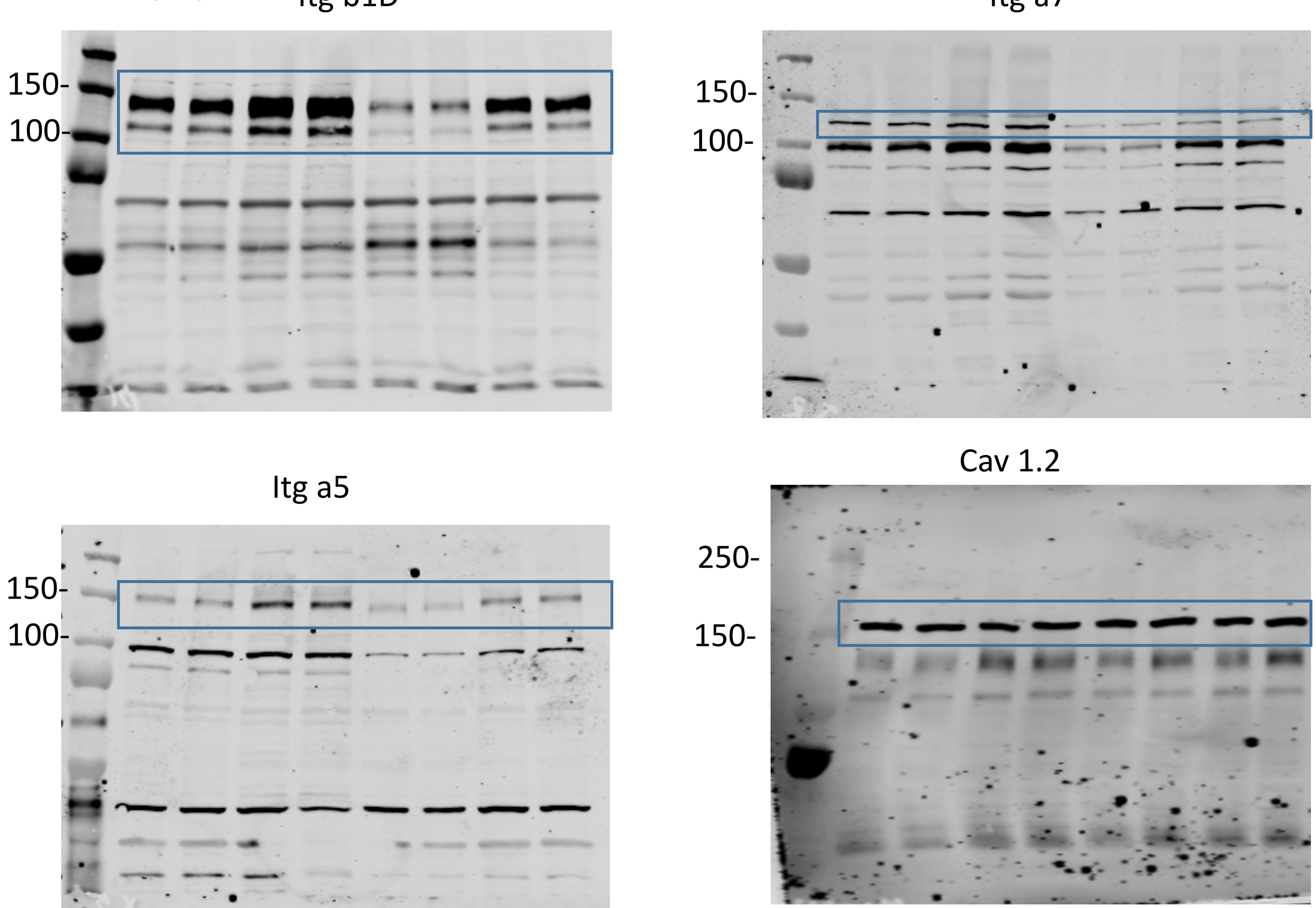

Supplementary Figure 9.

Uncropped blots Fig.6b

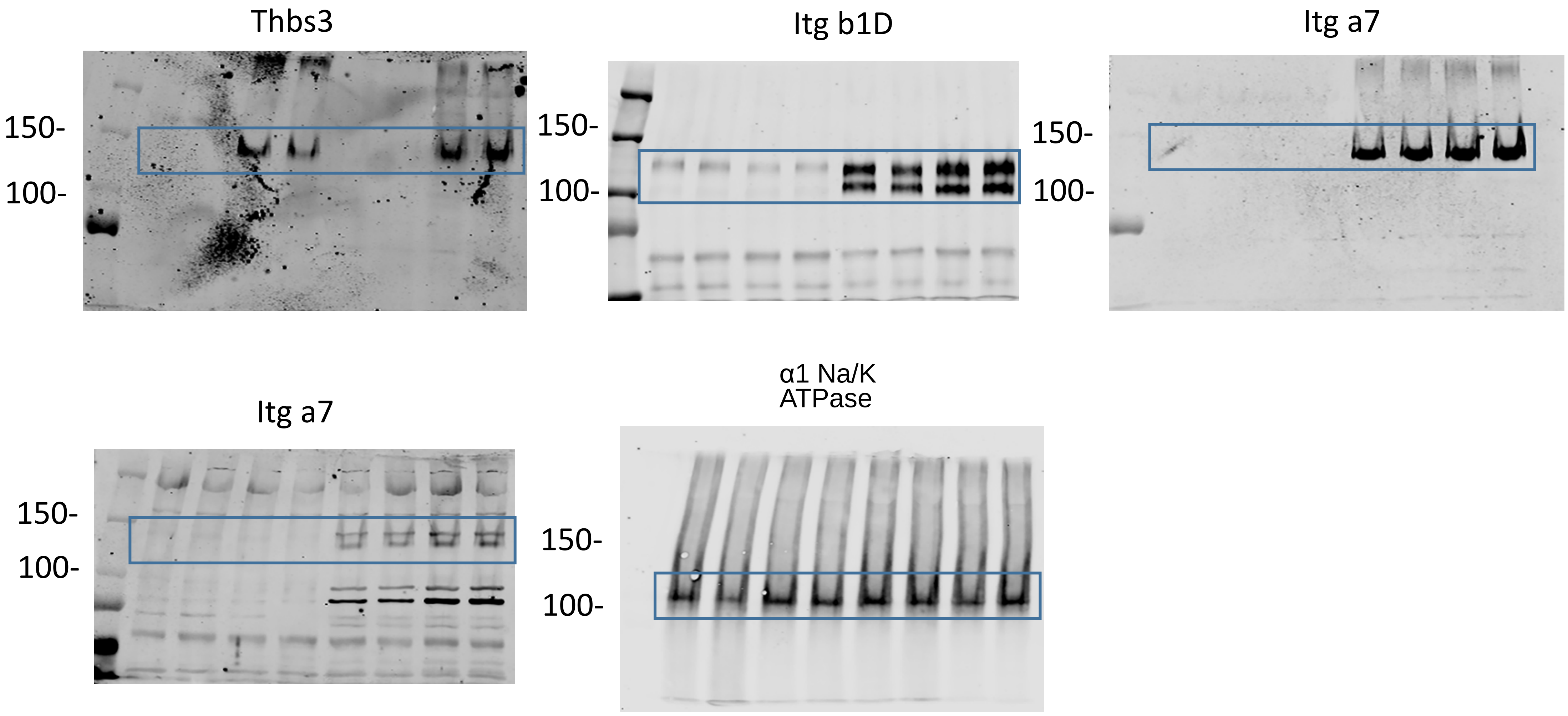

Supplementary Figure 9.

Uncropped blots Fig.7c

Lumican

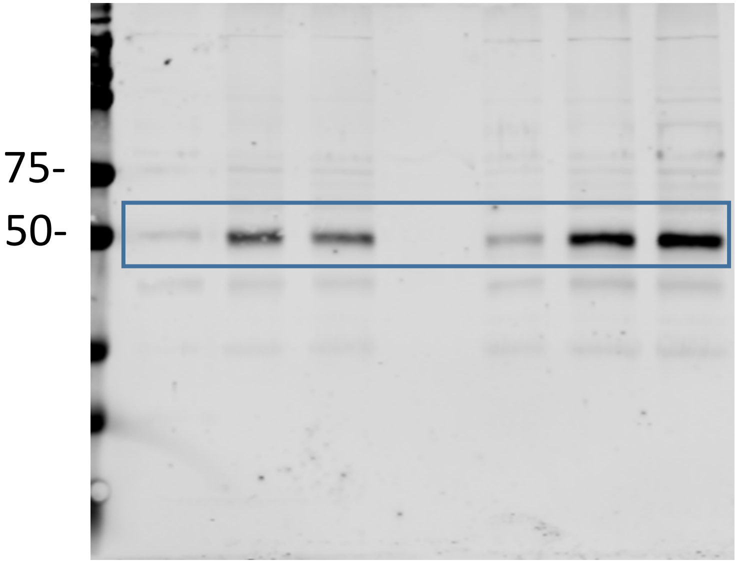

Fibronectin

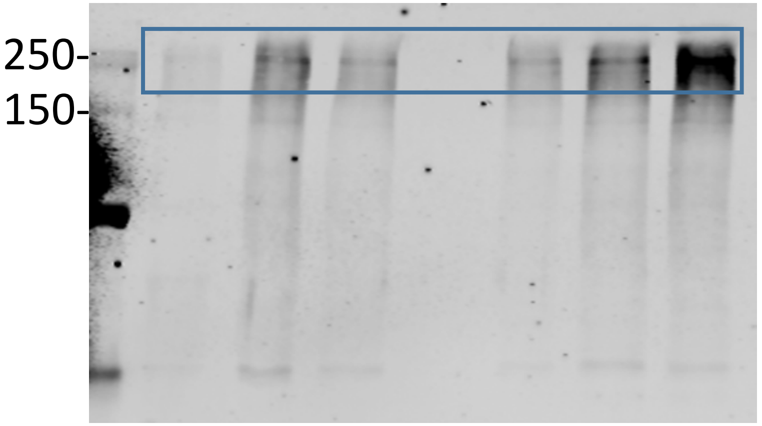

Fibromodulin

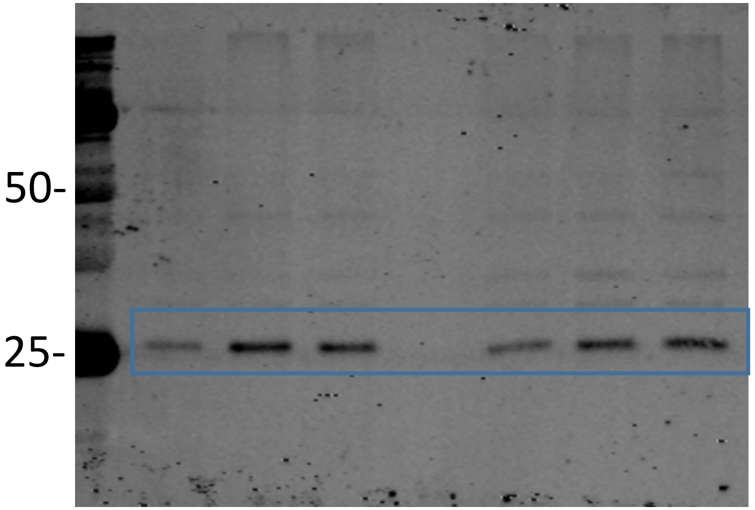

Collagen 4

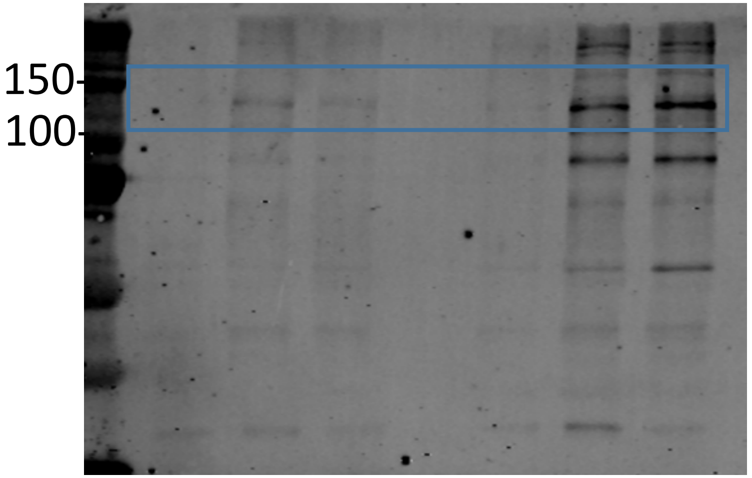

Silver Stain

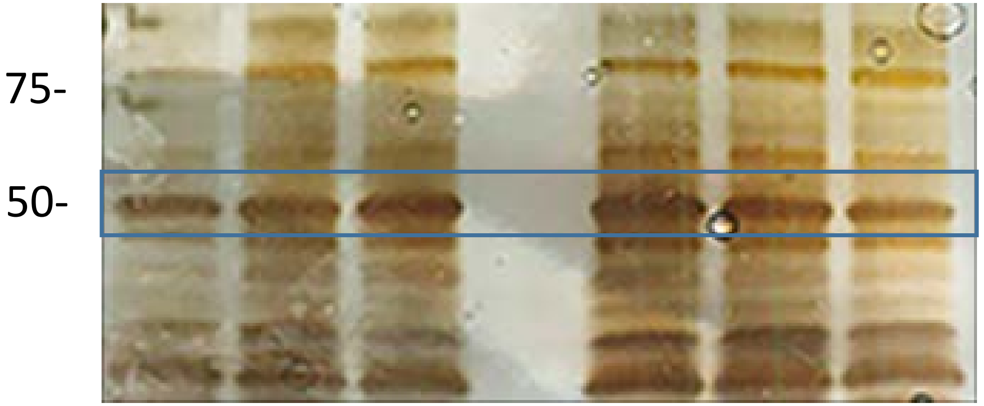

Supplementary Figure 9.

Uncropped blots Supplementary Fig.7b

Thbs3

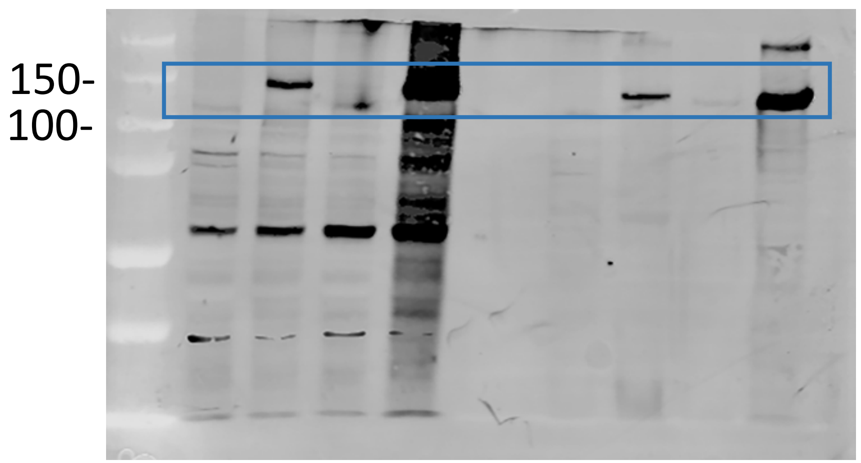

Gapdh

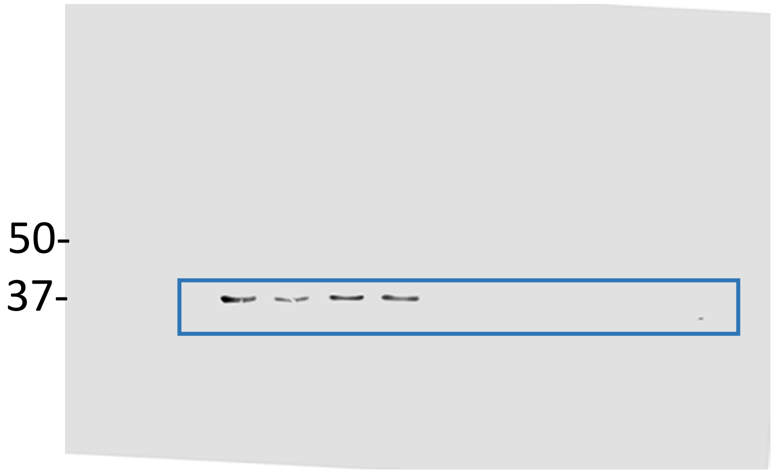

Thbs4

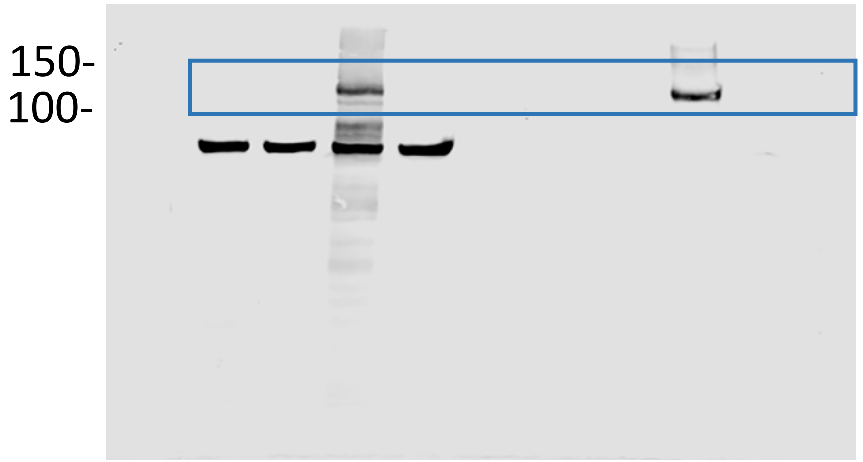

Supplementary Figure 9.

Uncropped blots Fig.8

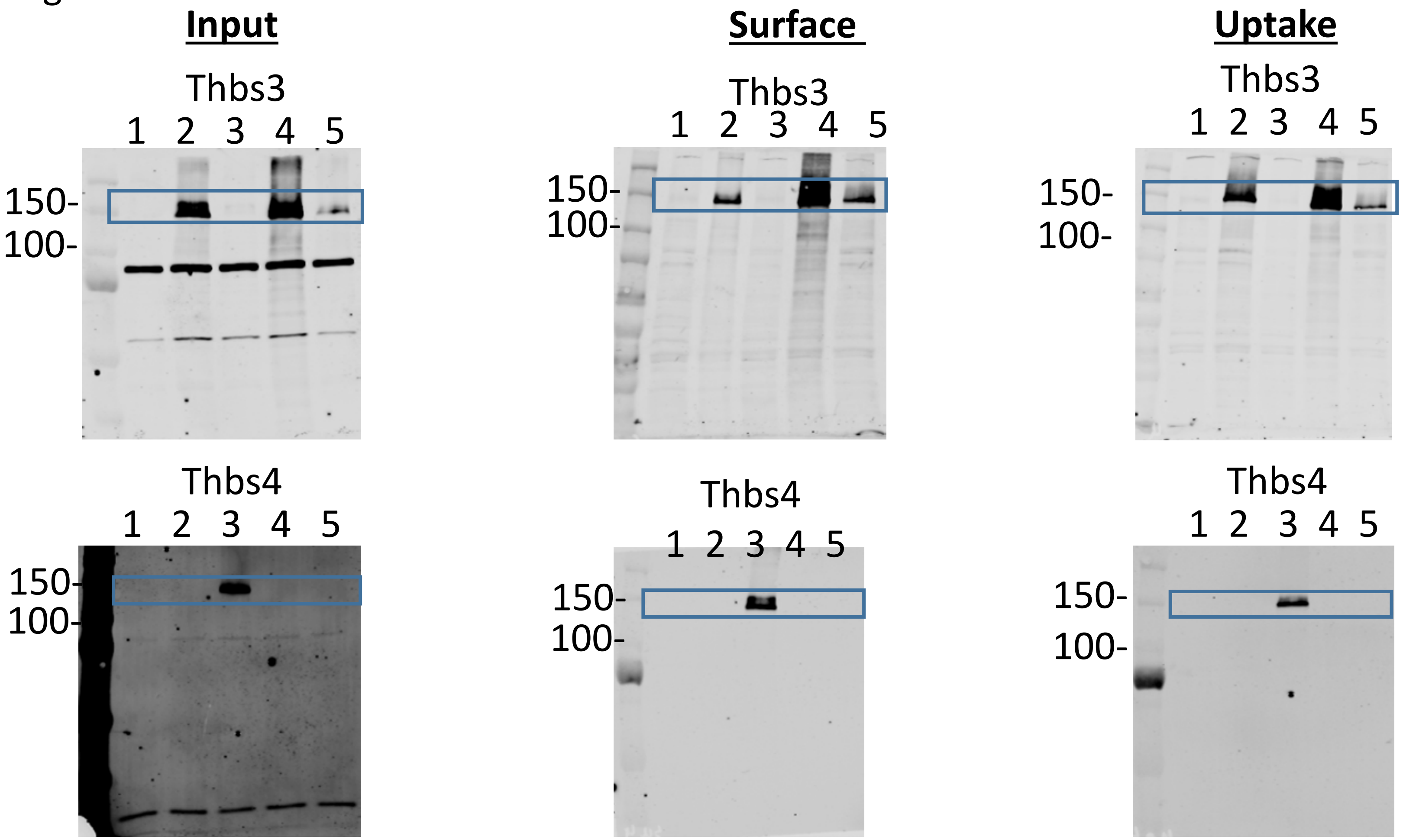

1 Ad B-Gal
2 Ad Thbs3
3 Ad Thbs4
4 Ad Thbs3 RGD
5 recomb. Thbs3

Not shown in primary figures but to demonstrate Thbs3 or Thbs4 overexpression byadenovriral or with recombinant protein

Supplementary Figure 9.

Uncropped blots Fig.8 used for quantification **Input**

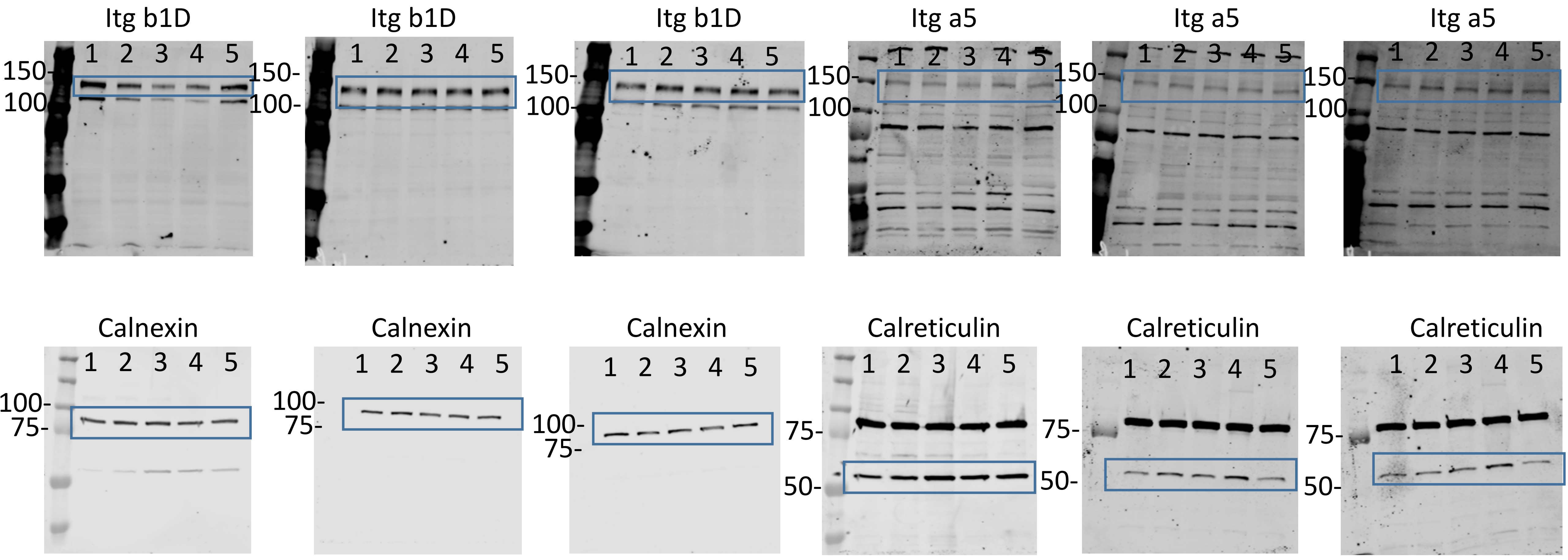

1 Ad B-Gal
2 Ad Thbs3
3 Ad Thbs4
4 Ad Thbs3 RGD
5 rec Thbs3

Supplementary Figure 9.

Uncropped blots Fig.8 used for quantification **Surface**

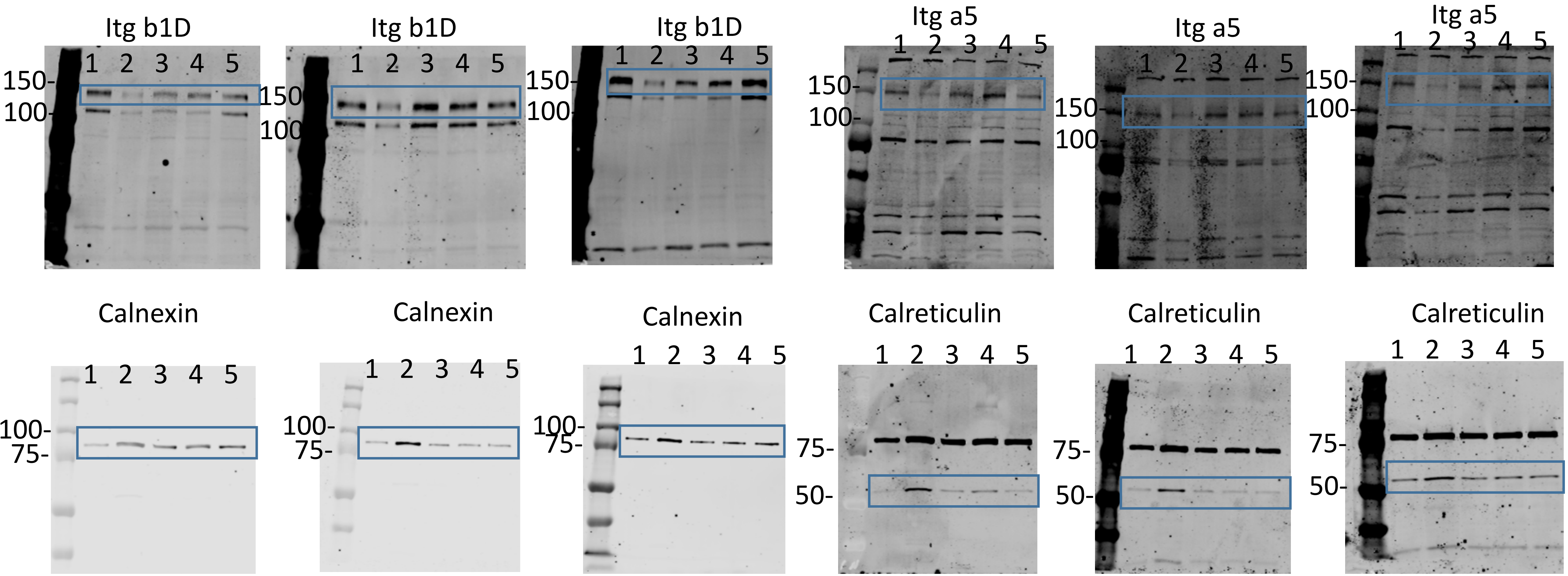

1 Ad B-Gal
2 Ad Thbs3
3 Ad Thbs4
4 Ad Thbs3 RGD
5 rec Thbs3

Supplementary Figure 9.

Uncropped blots Fig.8 used for quantification **Uptake**

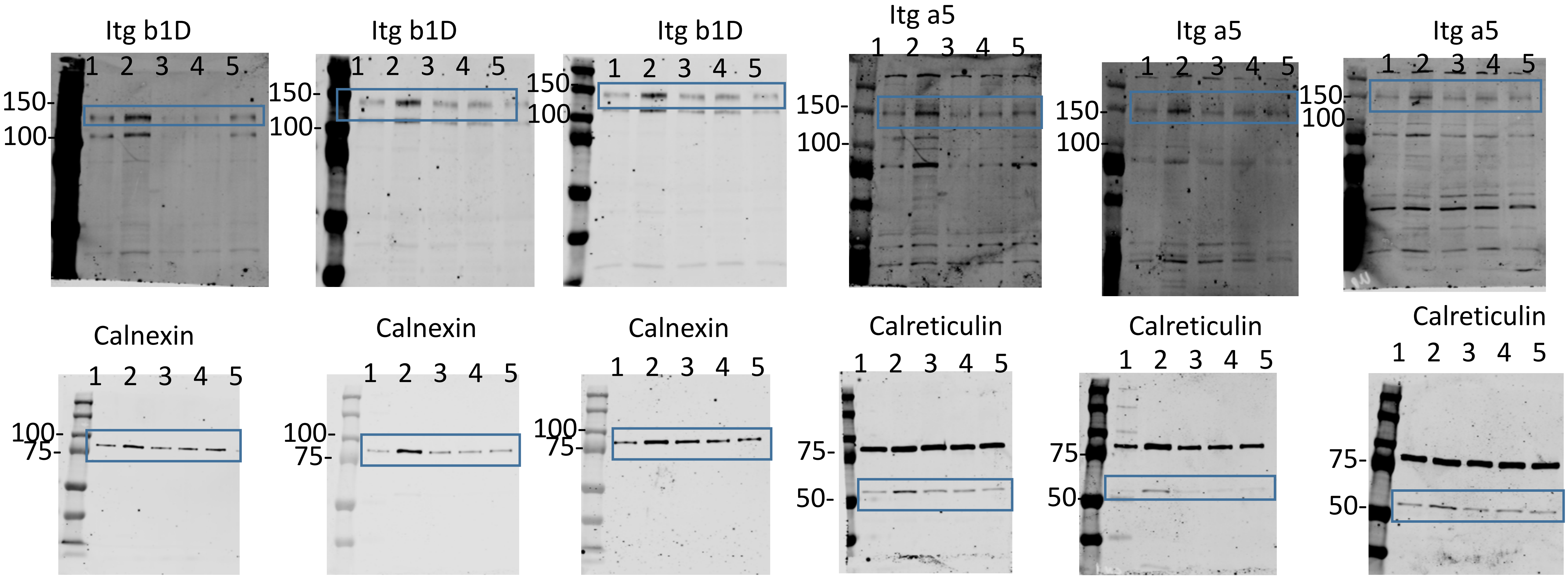

1 Ad B-Gal
2 Ad Thbs3
3 Ad Thbs4
4 Ad Thbs3 RGD
5 rec Thbs3

Supplemental Figure 9. The date over the last 20 pages of this supplemental figure are uncropped Western blot gel images for the indicated proteins with the migration size shown relative to molecular weight standards that are shown. Some of the data were only used in quantitation and were not actually western images shown in the manuscript
