# Supplementary material for: Thrombospondin-3 augments injury-induced cardiomyopathy by intracellular integrin inhibition and sarcolemmal instability
Source: Nat Commun. 2019 Jan 8;10:76. doi: 10.1038/s41467-018-08026-8 (PMC6325143; doi:10.1038/s41467-018-08026-8)
Supplement: Supplementary file 3 — Reporting Summary [file 41467_2018_8026_MOESM3_ESM.pdf]

nature research

Corresponding author(s): Jeffery D, Molkentin

Reporting Summary

Nature Research wishes to improve the reproducibility of the work that we publish. This form provides structure for consistency and transparency in reporting. For further information on Nature Research policies, see Authors & Referees and the Editorial Policy Checklist.

Statistical parameters

When statistical analyses are reported, confirm that the following items are present in the relevant location (e.g. figure legend, table legend, main text, or Methods section).

n/a	Confirmed	
☐	☒	The exact sample size (*n*) for each experimental group/condition, given as a discrete number and unit of measurement
☐	☒	An indication of whether measurements were taken from distinct samples or whether the same sample was measured repeatedly
☐	☒	The statistical test(s) used AND whether they are one- or two-sided<br>*Only common tests should be described solely by name; describe more complex techniques in the Methods section.*
☐	☒	A description of all covariates tested
☐	☒	A description of any assumptions or corrections, such as tests of normality and adjustment for multiple comparisons
☐	☒	A full description of the statistics including central tendency (e.g. means) or other basic estimates (e.g. regression coefficient) AND variation (e.g. standard deviation) or associated estimates of uncertainty (e.g. confidence intervals)
☐	☒	For null hypothesis testing, the test statistic (e.g. *F*, *t*, *r*) with confidence intervals, effect sizes, degrees of freedom and *P* value noted<br>*Give P values as exact values whenever suitable.*
☒	☐	For Bayesian analysis, information on the choice of priors and Markov chain Monte Carlo settings
☒	☐	For hierarchical and complex designs, identification of the appropriate level for tests and full reporting of outcomes
☒	☐	Estimates of effect sizes (e.g. Cohen's *d*, Pearson's *r*), indicating how they were calculated
☐	☒	Clearly defined error bars<br>*State explicitly what error bars represent (e.g. SD, SE, CI)*

Our web collection on statistics for biologists may be useful.

Software and code

Policy information about availability of computer code

Data collection	Data collection procedures are described in the Method section.<br>Software used:<br>Gen5 BioTek www.biotek.com<br>GraphPad Prism GraphPad Software www.graphpad.com<br>NIS-Elements AR Nikon Instruments www.nikoninstruments.com<br>Leica Application Suite Leica Microsystems www.leica-microsystems.com<br>Ionwizard Ionoptix www.ionoptix.com<br>Felix 1.1 Horiba www.horiba.com
Data analysis	Data analysis procedures are described in the Method section.<br>Software used:<br>Gen5 BioTek www.biotek.com<br>GraphPad Prism GraphPad Software www.graphpad.com<br>NIS-Elements AR Nikon Instruments www.nikoninstruments.com<br>Leica Application Suite Leica Microsystems www.leica-microsystems.com<br>Ionwizard Ionoptix www.ionoptix.com

Felix 1.1 Horiba www.horiba.com

For manuscripts utilizing custom algorithms or software that are central to the research but not yet described in published literature, software must be made available to editors/reviewers upon request. We strongly encourage code deposition in a community repository (e.g. GitHub). See the Nature Research guidelines for submitting code & software for further information.

Data

Policy information about availability of data

All manuscripts must include a data availability statement. This statement should provide the following information, where applicable:

- Accession codes, unique identifiers, or web links for publicly available datasets
- A list of figures that have associated raw data
- A description of any restrictions on data availability

Data available on request from the authors

Field-specific reporting

Please select the best fit for your research. If you are not sure, read the appropriate sections before making your selection.

☒ Life sciences ☐ Behavioural & social sciences ☐ Ecological, evolutionary & environmental sciences

For a reference copy of the document with all sections, see nature.com/authors/policies/ReportingSummary-flat.pdf

Life sciences study design

All studies must disclose on these points even when the disclosure is negative.

Sample size	The n numbers used in this study reflects the minimum number needed to achieve statistical significance based on experience and previous power analysis. Sample sizes were chosen based on extensive prior experience of the lab in characterizing cardiac injury models in the mouse (including observed post-operative surgical mortality rates for myocardial infarction and TAC surgery).
Data exclusions	Mice with pressure gradients of less than 45 mmHg and fractional shortening greater than 30% at 2 weeks post-TAC were excluded from the results, as this indicated an unsuccessful surgery. Animals that did not survive the day of surgery were excluded.
Replication	The results of all in vivo experiments were reproducible as shown across multiple animals (exact n values indicated in the text and figures) over multiple surgical cohorts. In vitro experimental findings were reproduced at least 3 times (exact n values indicated in the text and figures) and replication was successful.
Randomization	The animals were not randomized because they were genetically identical within groups. Both sexes of mice were used.
Blinding	Blinding was performed for some experimental procedures with mice, although blinding was not possible in every instance. Analysis of echocardiographic data, all histological analyses, live cell imaging was performed by investigators blinded to experimental treatment or procedure.

Reporting for specific materials, systems and methods

Materials & experimental systems

n/a	Involved in the study	
☐	☒	Unique biological materials
☐	☒	Antibodies
☐	☒	Eukaryotic cell lines
☒	☐	Palaeontology
☐	☒	Animals and other organisms
☒	☐	Human research participants

Methods

n/a	Involved in the study	
☒	☐	ChIP-seq
☒	☐	Flow cytometry
☒	☐	MRI-based neuroimaging

Unique biological materials

Policy information about availability of materials

Obtaining unique materials	Mus musculus, C57Bl/6J, tetO-Thbs3

Obtaining unique materials

pShuttle-CMV-Thbs3-WT
pShuttle-CMV-Thbs3-RGD
adThbs3-WT
adThbs3-RGD

Antibodies

Antibodies used

rabbit anti Armet Abcam Cat #ab67271
rabbit anti ATF6α Abcam Cat #ab37149
rabbit anti calreticulin (CRT) Cell Signaling Technology Cat #2891
rabbit anti calnexin (CXN) Abcam Cat #ab75801
rabbit anti GRP78/BiP Sigma-Aldrich Cat #G8918
rabbit anti PDI Cell Signaling Technology Cat #2446
mouse anti PDI Abcam Cat #ab2792
rabbit anti integrin α10 EMD Millipore Cat #AB6030
rabbit anti integrin α9 Abcam Cat #ab140599
rabbit anti integrin α7 Santa Cruz Biotechnology Cat #sc-27706
rabbit anti integrin α6 Cell Signaling Technology Cat #3750
rabbit anti integrin α5 EMD Millipore Cat #AB1928
rabbit anti integrin α4 EMD Millipore Cat #AB1924
mouse anti integrin α2 Lifespan Biosciences Cat #LS-C159934
rabbit anti integrin β3 Cell Signaling Technology Cat #4702
mouse anti Laminin2 Sigma Cat #L0063
mouse anti β1D-integrin EMD Millipore Cat # MAB1900
Mouse anti-c-Myc Santa Cruz Biotechnology Cat #sc40
mouse anti Thbs1 Lifespan Biosciences Cat #LS-B4155
mouse anti Thbs2 BD Bioscience Cat #611150
rabbit anti Thbs3 Proteintech Cat #19727-1-AP
rabbit anti Thbs4 Santa Cruz Biotechnology Cat #sc-7657
rabbit anti COMP (Thbs5) Proteintech Cat #13641-1-AP
rabbit pan-cadherin Cell Signaling Technology Cat #4068
rabbit anti Sodium Potassium ATPase Cell Signaling Technology Cat #ab76020
rabbit anti Cacnac1 Alomone Labs Cat #ACC-022
mouse anti β-Tubulin LI-COR Cat #926-42211
mouse anti Gapdh Fitzgerald Cat # 10R-G109A
goat anti-Mouse IRdye 800CW LI-COR Cat #926-32350
goat anti-Rat IRdye 800CW LI-COR Cat #926-32219
goat anti-Rabbit IRdye 800CW LI-COR Cat #926-32211
goat anti-Mouse IRdye 680RD LI-COR Cat #926-68072
goat anti-Rabbit IRdye 680RD LI-COR Cat #925-68073
goat anti-Mouse Alexa Fluor-488 ThermoFisher Scientific Cat #A11029
goat anti-Rabbit Alexa Fluor-488 ThermoFisher Scientific Cat #A11008
goat anti-Mouse Alexa Fluor-568 ThermoFisher Scientific Cat #A11031
goat anti-Rabbit Alexa Fluor-568 ThermoFisher Scientific Cat #A11036

Validation

Antibodies were validated by manufacturers, correct molecular weight, correct localization and overexpression or knockout samples whenever possible.

Eukaryotic cell lines

Policy information about cell lines

Cell line source(s)

COS-7 cells (ATCC, #CRL-1651)

Authentication

None of the cell lines were authenticated.

Mycoplasma contamination

The cell lines were not tested for mycoplasm contamination.

Commonly misidentified lines
(See ICLAC register)

No commonly misidentified lines were used in this study.

Animals and other organisms

Policy information about studies involving animals; ARRIVE guidelines recommended for reporting animal research

Laboratory animals

Only laboratory derived mice were used in this study. All experimental procedures with these mice were approved by the Institutional Animal Care and Use Committee of Cincinnati Children's Medical Center, protocols IACUC 2015-0047 and 2016-0069. We have complied with the relevant ethical considerations for animal usage overseen by this committee. The number of mice used in this study reflects the minimum number needed to achieve statistical significance based on experience and previous power analysis. Blinding was performed for some experimental procedures with mice, although blinding was not possible in every instance. Both sexes of mice were used.

Wild animals	No wild animals were used in this study.
Field-collected samples	Study did not involve samples collected in the field.
